# Hierarchical metabolic engineering for rewiring cellular metabolism

**DOI:** 10.1093/femsre/fuaf047

**Published:** 2025-09-27

**Authors:** Tiantian Chai, Yuxuan Tao, Chunlei Zhao, Xiulai Chen

**Affiliations:** School of Biotechnology and Key Laboratory of Industrial Biotechnology of Ministry of Education, Jiangnan University, Wuxi 214122, China; School of Biotechnology and Key Laboratory of Industrial Biotechnology of Ministry of Education, Jiangnan University, Wuxi 214122, China; School of Biotechnology and Key Laboratory of Industrial Biotechnology of Ministry of Education, Jiangnan University, Wuxi 214122, China; School of Biotechnology and Key Laboratory of Industrial Biotechnology of Ministry of Education, Jiangnan University, Wuxi 214122, China

**Keywords:** metabolic engineering, synthetic biology, cell factory, pathway rewiring, machine learning, enzyme engineering

## Abstract

Metabolic engineering is a key enabling technology for rewiring cellular metabolism to enhance production of chemicals, biofuels, and materials from renewable resources. However, how to make cells into efficient factories is still challenging due to its robust metabolic networks. To open this door, metabolic engineering has realized great breakthroughs through three waves of technological research and innovations, especially the third wave. To understand the third wave of metabolic engineering better, we discuss its mainstream strategies and examples of its application at five hierarchies, including part, pathway, network, genome, and cell level, and provide insights as to how to rewire cellular metabolism in the context of maximizing product titer, yield, and productivity. Finally, we highlight future perspectives on metabolic engineering for the successful development of cell factories.

## Teaser

Hierarchical metabolic engineering has enabled the efficient reprogramming of cellular metabolism to create microbial cell factories, offering a promising alternative for the green and sustainable production of chemicals.

## Introduction

Metabolic engineering is the science that seeks to improve product formation or cellular properties directly through modifying specific biochemical reactions or introducing new genes with recombinant DNA technology (Nerem 1991, Stephanopoulos 1994, Davy et al. 2017, Dasgupta et al. 2020). Metabolic engineering has provided an alternative way to replace as many petroleum-based refineries as possible through metabolically engineering organisms to produce useful chemicals and fuels from renewable biomass (Clomburg et al. 2017, Gong et al. 2017, Ko et al. 2020), such as 1,4-butanediol (Zhu et al. 2022b), artemisinin (Zhu et al. 2022a), succinic acid (Song et al. 2024), algal oil (Ajjawi et al. 2017), and poly(lactate-coglycolate) (Choi et al. 2016). The field of metabolic engineering has reached its present industrially proven level through several waves of technological research and innovations.

During the first wave of metabolic engineering, which started at the beginning of the decade of the 1990s, scientists recognized that natural pathways could be enumerated and assessed for converting a specific substrate to a target product. This rational understanding culminated with two seminal papers that essentially initiated the field of metabolic engineering (Bailey 1991, Stephanopoulos and Vallino 1991, Han et al. 2023). Most initial metabolic engineering efforts relied on such rational approaches to pathway analysis and flux optimization to regulate cellular metabolism and redirect flux to the desired products (Volk et al. 2023). A classic example is the overproduction of lysine in *Corynebacterium glutamicum*. When pyruvate carboxylase and aspartokinase were identified as possible bottlenecks by labeled glucose and flux analysis, the simultaneous expression of both enzymes increased flux both into and out of the Tricarboxylic acid cycle (TCA cycle) to balance the supply of intermediates, thus leading to a 150% increase in lysine productivity with the same growth rate as the control strain (Koffas et al. 2003, Sen 2024).

During the second wave of metabolic engineering, in the 2000s, initial systems biology technologies, typically genome-scale metabolic models (Edwards and Palsson 2000, , Madhavan et al. 2023, Gong et al. 2024), extended the view of metabolic pathways and approaches for their optimal functioning at the systemic level. This holistic understanding was boosted by Bernhard Ø Palsson to bridge a mechanistic genotype–phenotype relationship for exploring the metabolic potential of cell factory and identifying the target genes of metabolic engineering (Feist and Palsson 2008, O’Brien et al. 2015, Holbrook-Smith et al. 2024). This change expanded metabolic engineering to produce chemicals that are currently used as fuels, materials, and pharmaceutical ingredients (Nielsen 2017). Examples include that genome-scale *Saccharomyces cerevisiae* and *Escherichia coli* metabolic model predicted the strategies for the production of bioethanol (Bro et al. 2006, Yan et al. 2024) and adipic acid/hexamethylenediamine/6-aminocaproic acid (Burk et al. 2010, Das et al. 2020), respectively; flux scanning based on enforced objective flux identified the overexpression of key genes to enhance lycopene production (Shi et al. 2025); multiobjective memetic algorithm identified the key targets of gene knockout for the production of cubebol (Mischko et al. 2018), l-threonine (Du et al. 2020), and l-valine (Hao et al. 2022).

The third, and present, wave of metabolic engineering, started with the work of Jay D. Keasling in the 2010s. A complete metabolic pathway was designed, constructed, and optimized with synthetic nucleic acid elements for the production of natural–noninherent chemicals, such as artemisinin (Kim et al. 2020, Huang and Fang 2021). This event started the application of synthetic biology in the context of metabolic engineering. As synthetic biology become more advanced, their application will rapidly expand the array of attainable products, both natural/nonnatural and inherent/noninherent (Wang et al. 2024b, Xu et al. 2020), as well as the titers and rates at which they can be produced and even the specific organisms used for the task (Avci et al. 2025). Thus, synthetic biology endows remarkable new capabilities to organisms during the development of metabolic engineering. Currently, various cell factories are being developed to efficiently manufacture chemicals (Lee et al. 2019, Liao et al. 2020) (Table 1), including biofuels (Liu et al. 2021, Zhang et al. 2024b) such as 2-phenylethanol (Gao et al. 2023), commodity chemicals (Scown and Keasling 2022) such as cadaverine (Zhao et al. 2022), pharmaceuticals (Jung et al. 2024) such as opioids (Galanie et al. 2015), nutraceuticals such as myo-inositol (Gupta et al. 2017), antibiotic such as lactams (Lee et al. 2025), anticancer drug such as vinblastine (Zhang et al. 2022), vaccine adjuvant (Martin et al. 2024) such as QS-21 (Liu et al. 2024b), nonnatural amino acids such as pazamine (Sosa et al. 2024), and alkaloids such as psilocybin (Huang et al. 2025).

**Table 1.** Representative chemicals produced through hierarchical metabolic engineering.

Chemicals	Hosts	Fermentation parameters	Metabolic engineering strategies	References
Bulk chemicals				
Organic acids				
3-Hydroxypropionicacid	*K. phaffii*	27.0 g/l, 0.19 g/g—methanol, 0.56 g/l/h	• Transporter engineering • Tolerance engineering • Chassis engineering	Àvila-Cabré et al. (2025 )
	*S. cerevisiae*	18 g/l, 0.17 g/g—glucose	• Enzyme engineering • Cofactor engineering	Tong et al. (2021)
	*C. glutamicum*	62.6 g/l, 0.51 g/g—glucose	• Substrate engineering • Genome editing engineering	Chen et al. (2017a)
Lactic acid	*C. glutamicum*	l-lacticacid: 212 g/l, 97.9 g/g—glucose d-lacticacid: 264 g/l, 95.0 g/g—glucose	• Modular pathway engineering	Tsuge et al. (2019)
Acetic acid	*E. coli*	20.09 g/l, 0.52 g/g—glucose	• Substrate engineering • Modular pathway engineering	Zhu et al. (2024)
Pyruvic acid	*L. lactis*	54.6 g/l	• Substrate engineering • Chassis engineering	Suo et al. (2021)
Propionic acid	*P. freudenreichii*	136.23 g/l, 0.5 g/g—glucose, 0.57 g/l/h	• Modular pathway engineering	Chen et al. (2013)
Butyric acid	*E. coli*	29.8 g/l	• Modular pathway engineering • Genome editing engineering • Signaling transplant engineering	Guo et al. (2020a)
Glycolate	*E. coli*	52.2 g/l	• Cofactor engineering • Modular pathway engineering	Yang et al. (2024)
Fumaric acid	*A. pullulans*	93.9 g/l	• Genome editing engineering • Modular pathway engineering	Wei et al. (2023)
Glycolic acid	*E. coli*	2.25 g/l, 0.51 g/g—xylose	• Modular pathway engineering • Substrate engineering	Cabulong et al. (2021)
Malonic acid	*Y. lipolytica*	63.6 g/l, 0.41 g/l/h	• Modular pathway engineering • Genome editing engineering • Substrate engineering	Yang et al. (2025)
Succinic acid	*C. glutamicum*	10.85 g/l	• Cofactor engineering • Modular pathway engineering • Chassis engineering	Liang et al. (2025b)
	*E. coli*	153.36 g/l, 2.13 g/l/h	• Modular pathway engineering • High-throughput genome engineering • Codon optimization	Pan et al. (2024)
Itaconic acid	*S. cerevisiae*	1.2 g/l	• Promoter engineering • Transporter engineering	Xu et al. (2025)
Muconic acid	*C. glutamicum*	54 g/l, 0.197 g/g—glucose, 0.34 g/l/h	• Modular pathway engineering • Chassis engineering	Lee et al. (2018)
Amino acids			•	
Lysine	*C. glutamicum*	223.4 g/l, 0.68 g/g—glucose	• Cofactor engineering • Transporter engineering • Promoter engineering	Liu et al. (2023)
Valine	*E. coli*	59 g/l, 0.39 g/g—glucose	• Transcription factor engineering • Cofactor engineering • Genome editing engineering	Gao et al. (2024)
Isoleucine	*E. coli*	56.6 g/l, 1.66 g/l/h	• Transporter engineering • Chassis engineering	Zhang et al. (2025)
Tryptophan	*E. coli*	52.1 g/l, 0.171 g/g—glucose, 1.45 g/l/h	• Promoter engineering • Transporter engineering • Modular pathway engineering	Guo et al. (2021)
Threonine	*E. coli*	118.2 g/l, 0.57 g/g—glucose, 2.46 g/l/h	• Substrate engineering • Signaling specificity engineering	Song et al. (2024)
Tyrosine	*E. coli*	109.2 g/l, 0.292 g/g—glucose, 2.18 g/l/h	• Compartmentalization engineering • Transporter engineering • Modular pathway engineering	Chen et al. (2025b)
Arginine	*E. coli*	132 g/l, 0.51 g/g—glucose, 2.75 g/l/h	• Signaling transplant engineering • High-throughput genome engineering • Modular pathway engineering	Jiang et al. (2023)
Alcohols			•	
1,3-Propanediol	*E. coli*	86.6 g/l, 0.84 g/g—glucose	• Modular pathway engineering • Cofactor engineering • Genome editing engineering	Lee et al. (2025)
1,2-Propanediol	*E. coli*	1.48 g/l	• Substrate engineering • Modular pathway engineering	Nonaka et al. (2021)
1,4-Butanediol	*E. coli*	1.5 g/l	• Enzyme engineering • Cofactor engineering	Ni et al. (2024)
1,3-Butanediol	*E. coli*	23 g/l, 0.25 g/g—glucose	• Cofactor engineering • Transcription factor engineering • Promoter engineering	Islam et al. (2023)
2,3-Butanediol	*C. glutamicum*	144.9 g/l	• Codon optimization • Cofactor engineering	Mattanovich et al. (2021)
1,5-Pentanediol	*C. glutamicum*	43.4 g/l	• Enzyme engineering • Cofactor engineering • Modular pathway engineering	Sohn et al. (2024)
Amines			•	
Isobutylamine	*E. coli*	10.67 g/l	• Modular pathway engineering	Kim et al. (2021)
1,3-Diaminopropane	*E. coli*	13 g/l, 0.1 g/g—glucose,0.19 g/l/h	• High-throughput genome engineering • Modular pathway engineering	Chae et al. (2015)
Putrescine	*E. coli*	76 g/l	• Substrate engineering • Modular pathway engineering	Wang et al. (2024)
1,5-Diaminopentane	*C. glutamicum*	103.8 g/l, 0.31 g/g—glucose, 1.47 g/l/h	• Promoter engineering • Transporter engineering	Kim et al. (2018)
Cadaverine	*E. coli*	64.03 g/l, 0.23 g/g-glucose, 1.33 g/l/h	• Signaling transplant engineering • Modular pathway engineering	Liu et al. ( 2024a)
2-Phenylethanol	*R. toruloides*	1.06 g/l, 22.5 mg/g—glucose, 8 mg/l/h	• Cofactor engineering • Substrate engineering	Zheng et al. (2024)
Oleo-chemicals				
Oleoylethanolamide	*S. cerevisiae*	8115.7 µg/l, 405.8 µg/g—glucose	• Spatial substrate channel engineering • Enzyme engineering	Liu et al. (2020b)
Triacylglycerol	*S. cerevisiae*	1.76 g/l, 0.088 g/g—glucose	• Chassis engineering • Modular pathway engineering	Ferreira et al. (2018)
Docosahexaenoic acid	*M. alpina*	1.2 g/l	• Substrate engineering • Enzyme engineering	Tang et al. (2018)
Arachidonic acid	*M. alpina*	7.6 g/l	• Substrate engineering • Modular pathway engineering • Cofactor engineering	Hao et al. (2016)
Docosahexaenoic acid	*A. limacinum*	38.3 g/l	• Substrate engineering • Modular pathway engineering • Chassis engineering	Ren et al. (2017)
Triacylglycerol	*R. toruloides*	0.30 g/g—glucose	• Chassis engineering	Arbter et al. (2019)
Docosapentaenoic acid	*Schizochytrium sp*	18.7%	• Modular pathway engineering • Promoter engineering • High-throughput genome engineering	Ren et al. (2015)
Natural products				
Terpenoids				
Farnesene	*Y. lipolytica*	14.7 g/l β-farnesene, 46 mg/g—methanol	• Promoter engineering • Cofactor engineering • Modular pathway engineering	Li et al. (2024)
	*Y. lipolytica*	57.08 mg/l α-farnesene	• Codon optimization • Modular pathway engineering	Yang et al. (2016)
Squalene	*Y. lipolytica*	514.33 mg/l	• Cofactor engineering • Modular pathway engineering	Lin et al. (2025)
Artemisinin	*S. cerevisiae*	3.97 g/l	• Enzyme engineering • Modular pathway engineering	Guo et al. (2025)
Amorphadiene	*E. coli*	30 g/l	• Chassis engineering • Modular pathway engineering	Shukal et al. (2019)
Taxadiene	*E. coli*	1.02 g/l	• Modular pathway engineering	Ajikumar et al. (2010)
Geraniol	*E. coli*	2 g/l	• Modular pathway engineering	Liu et al. (2016)
Coniferyl alcohol	*E. coli*	124.9 mg/l	• Modular pathway engineering • Chassis engineering	Chen et al. (2017b)
Caffeylalcohol	*E. coli*	854.1 mg/l	• Modular pathway engineering • Chassis engineering	Chen et al. (2017b)
Alkaloids			•	
Paclitaxel	*S. cerevisiae*	0.97 µg/l	• Enzyme engineering • Cofactor engineering • Modular pathway engineering	Liang et al. (2025a)
Vincristine	*K. phaffii*	1.5 g/l	• Enzyme engineering • Modular pathway engineering	Gao et al. (2023)
Polyphenols	Polyphenols	Polyphenols		
Resveratrol	*E. coli*	2.34 g/l	• Enzyme engineering • Promoter engineering	Feng et al. (2022)
Vanillin	*E. coli*	24.7 mg/l	• Modular pathway engineering	Ni et al. (2015)
Ferulic acid	*E. coli*	5.09 g/l	• Cofactor engineering • Modular pathway engineering	Zhou et al. (2022)
Curcumin	*E. coli*	696.2 mg/l	• Enzyme engineering • Cofactor engineering • Transporter engineering	Chen et al. (2023)
Flavonoids				
Hesperidin	*B. subtilis*	2.70 g/l	• Promoter engineering	Zhou et al. (2023)
Baicalin	*Y. lipolytica*	346 mg/l	• Modular pathway engineering	Wang et al. (2022)
Quercetin	*B. subtilis*	35.6 g/l	• Substrate engineering • Modular pathway engineering	Niu et al. (2024)
Flaviolin	*E. coli*	26.0 mg/l	• Enzyme engineering • Signaling transplant engineering • Modular pathway engineering	Yang et al. (2018)
Carotenoids and derivatives			•	
Lycopene	*S. cerevisiae*	2.3 g/l	• Spatial substrate channel engineering	Larroude et al. (2017)
β-Carotene	*Y. lipolytica*	6.5 g/l	• Promoter engineering	Li et al. (2020)
Zeaxanthin	*E. coli*	722.46 mg/l, 23.16 mg/g DCW	• Modular pathway engineering	Yang and Guo (2014)
Astaxanthin	*E. coli*	432 mg/l, 9.62 mg/l/h	• Enzyme engineering • Spatial substrate channel engineering	Kang et al. (2019)
Retinol	*Y. lipolytica*	5.4 g/l	• Enzyme engineering • Modular pathway engineering	Ren et al. (2024)
Phenylpropanoids and derivatives			•	
Olivetolicacid	*E. coli*	80 mg/l	• Promoter engineering • Genome editing engineering	Stevens et al. (2013)
Others			•	
Rosmarinicacid	*E. coli*	172 mg/l	• Modular pathway engineering • Chassis engineering	Li et al. (2019)
Acetoin	*S. cerevisiae*	97.5 g/l, 1.81 g/l/h	• Cofactor engineering • Enzyme engineering • Substrate engineering	Wang et al. (2025)
Aloesone	*E. coli*	30.9 mg/l	• Enzyme engineering • Signaling transplant engineering • Modular pathway engineering	Yang et al. (2018)
Biofuels				
Propanol	*P. freudenreichii* *C. beijerinckii*	12 g/l	• Chassis engineering	Hocq and Sauer (2022)
2-Propanol	*C. glutamicum*	4.91 g/l	• Codon optimization • Modular pathway engineering	Shi et al. (2024)
Butanol	*C. acetobutylicum*	14.3 g/l, 0.40 g/l/h	• Substrate engineering • Morphology engineering	Chen et al. (2025a)
Isobutanol	*S. oneidensis*	1321 mg/l, 0.394 g/g—lactic acid	• Signaling dynamics engineering • Genome editing engineering	Yu et al. (2025)
Isopentanol	*C. glutamicum*	2.76 g/l, 0.1 g/g, 0.058 g/l/h	• Enzyme engineering • Modular pathway engineering	Runguphan et al. (2021)
Isoprene	*S. cerevisiae*	3.7 g/l, 22.9 mg/g—glucose, 39 mg/l/h	• Enzyme engineering • Promoter engineering • Transcription factor engineering	Wang et al. (2017)
Isoamyl alcohol	*S. cerevisiae*	561 mg/l	• Promoter engineering • Transporter engineering • Compartmentalization engineering	Yuan et al. (2016)
Pentane	*Y. lipolytica*	4.98 mg/l	• Modular pathway engineering • Substrate engineering	Blazeck et al. (2013)
Hexanal	*Y. lipolytica*	600 mg/l	• Cofactor engineering	Santiago-Gómez et al. (2009)
Decanol	*Y. lipolytica*	550 mg/l	• Modular pathway engineering • Enzyme engineering	Rutter and Rao (2016)
Limonene	*S. cerevisiae*	2.63 g/l	• Cofactor engineering • Compartmentalization engineering	Kong et al. (2023)
Free fatty acids	*E. coli*	35.1 g/l, 0.84 g/l/h	• Cofactor engineering • High-throughput genome engineering	Fang et al. (2025)
Pharmaceuticals			•	
Opioids	*S. cerevisiae*	6.4 µg/l thebaine 0.3 µg/l hydrocodone	• Enzyme engineering • Modular pathway engineering	Galanie et al. (2015)
Thebaine	*E. coli*	2.1 mg/l	• Modular pathway engineering • Chassis engineering	Nakagawa et al. (2016)
Hydrocodone	*E. coli*	0.36 mg/l	• Modular pathway engineering • Chassis engineering	Nakagawa et al. (2016)
Oxytetracycline	*E. coli*	2.0 mg/l	• Promoter engineering • Genome editing engineering	Stevens et al. (2013)
Qs-21	*S. cerevisiae*	0.0012% w/w QS-21	• Compartmentalization engineering • Modular pathway engineering	Liu et al. (2024b), Martin et al. (2024)

**Figure 1.**
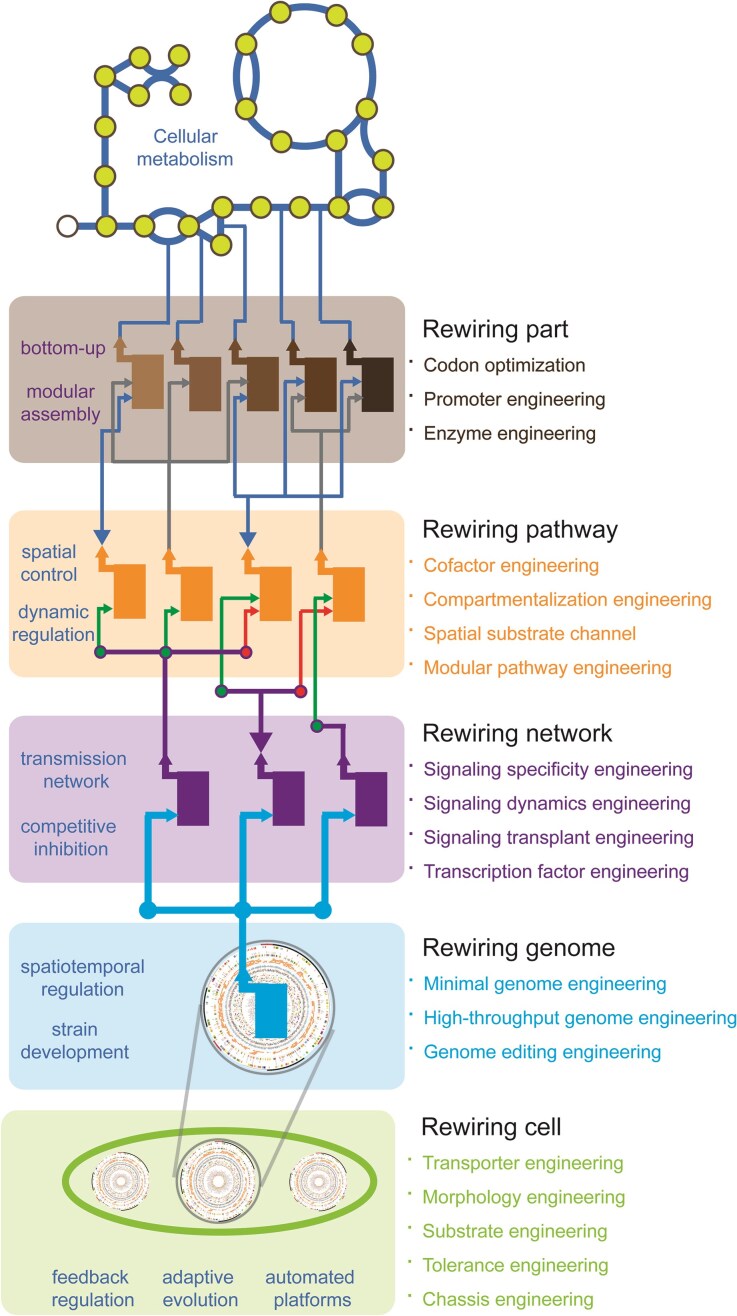
Hierarchical metabolic engineering for rewiring cellular metabolism in the third wave.

In this review, we focus our attention on the rapidly emerging strategies in the third wave of metabolic engineering for rewiring cellular metabolism at five hierarchies, including part, pathway, network, genome, and cell level (Fig. 1). We use several successful case studies to illustrate these ideas, and provide guidance as to when each of these strategies will be considered and what each of these strategies will be adopted. Finally, we offer future perspectives on how this field might evolve and what remaining challenges are left to be faced.

Cell factories can be engineered on the basis of genetic hierarchy control: (i) rewiring part through codon optimization, promoter engineering, and enzyme engineering; (ii) rewiring pathway through cofactor engineering, compartmentalization engineering, spatial substrate channel, and modular pathway engineering; (iii) rewiring network through signaling specificity engineering, signaling dynamics engineering, signaling transplant engineering, and transcription factor engineering; (iv) rewiring genome through minimal genome engineering, high-throughput genome engineering, and genome editing engineering; and (v) rewiring cell through transporter engineering, morphology engineering, substrate engineering, and tolerance engineering. Importantly, these hierarchical levels are not independent but dynamically interconnected: genome minimization constrains network complexity, modular pathway design must function in global regulatory circuits, and cell phenotypes emerge from the integration of rewiring at the part, pathway, network, and genome levels. Conversely, systemic challenges such as metabolic burden, regulatory feedback, and evolutionary instability influence these layers, affecting design choices and the long-term stability of engineered systems.

## Level 1: rewiring part

**Figure 2.**
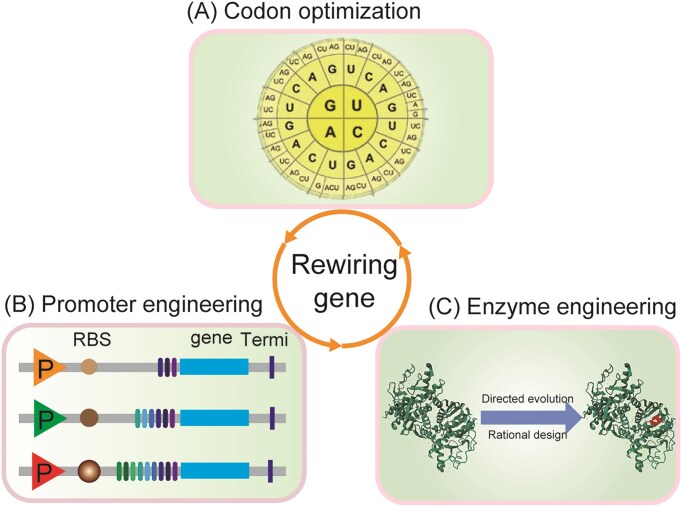
Overview of strategies for rewiring part. (A) Codon optimization for optimizing genetic code; (B) promoter engineering for tuning gene expression; and (C) enzyme engineering for improving enzyme catalytic efficiency.

Biological systems can be rationally reprogrammed by controlling their fundamental units—genes—which serve as the entry point for synthetic rewiring. As the most basic layer of metabolic engineering, gene-level modulation establishes the foundation for a partially connected higher-order network design. Genes can act as functional points in engineering biological systems for biosynthesis of many industrial chemicals. In this process, metabolic engineering is required for modifying metabolic pathways, which are involved to introduction of several genes. However, product titers and yields are often limited by one of these genes, which plays a critical role in improving the balance of key pathways for maximizing production of desired chemicals. To solve these problems, several strategies have shown great promise in generating a dynamic range for gene expression, including codon optimization, promoter engineering, and enzyme engineering (Fig. 2).

### Codon optimization

Codon optimization describes a gene engineering approach for improving the RNA and protein levels of a recombinant gene (Bijukumar and Somvanshi 2024), which use synonymous codon to optimize its codon composition without altering amino acid sequence. Several strategies have been developed to quantify codon usage and implement codon change, such as adopting the most optimal codons, adjusting the proportion of codon usage, replacing rare codons, and genetic code expansion.

Adjusting the proportion of codon usage involves globally aligning the codon composition of heterologous genes with that of highly expressed host genes, aiming to fine-tune mRNA stability, translation dynamics, and cotranslational folding, thereby ensuring efficient and reliable protein expression. (Mauro and Chappell 2014, Chin 2017). A notable instance for rational modulation of codon usage involves the strategic increase of adenine (A) nucleotide frequency at codon positions two to six, which contributes to enhancing thermodynamic stability of proteins by optimizing ribosome-binding efficiency. To enhance protein stability and thereby increase succinic acid production, a protein stability regulator based on a subset of codon combinations (ccsPSR) was developed by optimizing the codon usage of the *ribF* gene. The engineered strain *E. coli* FY-65 was able to produce 153.36 g/l succinic acid with its productivity up to 2.13 g/l/h. This methodology offers valuable insights for applications in synthetic biology that necessitate adjustable protein stability for industrial bioproduction (Pan et al. 2024).

The utilization of rare codons pertains to the deliberate incorporation of infrequently occurring codons within coding sequences, aimed at regulating the rate of translation and enhancing the efficiency of protein synthesis (Zhang et al. 2017). A significant illustration of this approach is the implementation of a rare codon-based screening strategy for identifying strains with high capability for l-lysine production. By developing an artificial rare codon system, the lysine codons within the tRNA promoter of *C. glutamicum* were substituted with the rare codon AAA. The resulting mutant strain *C. glutamicum* W2 exhibited a 9.7% enhancement in l-lysine production compared to that of wild-type strain (Yang et al. 2023).

The expansion of the genetic code, which involves the incorporation of noncanonical amino acids (ncAAs) into the central dogma, has established essential methodologies for the investigation and modification of biological processes (Chin 2014, Costello et al. 2024). A notable example of genetic code expansion is the reassignment of sense codons, which facilitates viral resistance and the production of encoded polymers. For the synthesis and reencoding of the genome, *E. coli* syn61 was constructed by replacing two normal codons (serine codons TCG and TCA) and one termination codon (TAG) with synonymous codons. After adaptive evolution, *E. coli* syn61 exhibited complete resistance to the virus mixture. Subsequently, by reassigning each codon to several ncAAs, *E. coli* syn61 was possible to effectively synthesize proteins containing three different ncAAs and nonclassical polymers and large rings (Robertson et al. 2021).

Codon optimization has evolved from a basic substitution approach into a versatile engineering strategy for the precise regulation of gene expression. By adjusting codon usage, rare codon distribution, and expanding the genetic code, diverse optimization strategies enable precise control over translation efficiency and functional output. Despite these advances, rational codon design remains constrained by an incomplete understanding of sequence–structure–function relationships and the complex, context-dependent nature of translational regulation. In addition, many optimization strategies are still guided by host-specific empirical rules, limiting their generalizability and potentially reducing their effectiveness when applied in different organisms or expression systems. In industrial applications, this technology can improve the production efficiency of chemicals, demonstrating its practical potential. Nevertheless, codon modifications can unintentionally perturb mRNA secondary structures, affect translation kinetics, or interfere with cotranslational folding, highlighting the need for more integrative and predictive design frameworks. Future research should focus on developing predictive tools and orthogonal systems to broaden the applicability of this approach in intricate biosynthetic frameworks (Klump et al. 2020, Zabolotskii et al. 2023).

### Promoter engineering

Promoter engineering is a powerful tool for modulating promoter transcriptional capacity by mutating, enhancing, or altering promoter DNA sequence to generate the dynamic range, which is essential for fine-tuning gene expression in metabolic engineering applications. Numerous advancements in promoter engineering mainly focus on the design of hybrid promoters, the development of synthetic promoter scaffolds and libraries, the exploration of intergenic regions, and the optimization of ribosome binding sites (RBS) (Cazier and Blazeck 2021).

The design of hybrid promoters conceptualizes sequence promoter elements as modular components that can be rearranged, exchanged, or duplicated. These modular elements encompass one or more transcription factor binding site DNA sequences, thereby enhancing promoter strength or facilitating the establishment of novel transcriptional regulatory patterns, particularly those that are inducible (Deaner and Alper 2018). A representative case of hybrid promoter engineering involves the application of portable 5′-UTR secondary structures to augment protein expression in *Bacillus licheniformis*. In this study, a hybrid promoter engineering strategy was implemented in *B. licheniformis* DW2 to enhance mRNA stability and facilitate translation initiation. Specifically, a hairpin structure was incorporated into the 5′-UTR to serve as an mRNA stabilizer, while a fully mismatched Shine–Dalgarno (SD) sequence was integrated within the hairpin loop to improve translation efficiency. This hybrid promoter strategy could enhance the efficiency of gene translation by optimizing mRNA folding free energy, which showed good compatibility with various target proteins, such as nattokinase and keratinase (Xiao et al. 2020).

Synthetic promoter engineering involves the deliberate design and assembly of promoter sequences through the integration of core promoter elements, regulatory motifs, and untranslated regions. This approach seeks to facilitate precise and adjustable regulation of gene transcription (Garg et al. 2012). A significant instance of synthetic promoter engineering is the utilization of synthetic promoters to establish a highly efficient expression system in *Bacillus subtilis*. To develop a more stable expression cassette, 10high-strength promoters were evaluated through transcriptomic analysis and promoter engineering techniques. The most potent promoter underwent further enhancement through the optimization of various elements, including spacer length, promoter sequence, SD sequence, binding sites for regulatory factors, and the terminator region. Consequently, the most effective expression cassette PSDP-4 was successfully developed, demonstrating a 3.84-fold increase in strength compared to that of the original promoter. Utilizing this cassette, a highly efficient expression system for *B. subtilis* DB104 was constructed, facilitating rapid, inducer-free protein expression within a 24 h timeframe (Jun et al. 2023).

The RBS plays a crucial role in determining both the abundance and quality of proteins by affecting the fidelity and efficiency of translation (Faure et al. 2016). An illustrative instance of RBS engineering is the development of an RBS library for modulating protein expression levels in *Bacillus* species. Given the absence of standardized and reliable genetic tools for these microorganisms, a synthetic hairpin RBS (shRBS) library was created to precisely regulate protein expression by incorporating a hairpin structure and a poly(A) sequence into the spacer region of the shRBS. This library facilitated a broad expression gradient of up to 10 000-fold and could be integrated with other gene expression regulatory elements, such as inducible promoters, to optimize more intricate cellular phenotypes and enable the regulation of metabolic pathways (Rao et al. 2024).

Promoter engineering has emerged as a critical approach for the optimization of gene expression in microbial hosts, facilitating applications in pathway balancing and enhanced protein production (Xu et al. 2019). Despite these advances, there still exists some issues, such as leaky expression, limited dynamic range, and incomplete understanding of promoter regulatory logic. Moreover, synthetic promoters often exhibit unpredictable behaviors in various contexts, including host strains, growth conditions, or chromosomal locations, thereby limiting their reliability and adaptability. Thus, future progress in promoter engineering will hinge on developing broad-spectrum promoters, elucidating regulatory promoter mechanisms, and integrating these insights with machine learning for predictive modeling and rational design (Sun et al. 2022). Establishing standardized promoter architectures and developing design principles that consider specific contexts will be crucial for expanding their utility in complex biosynthetic networks.

### Enzyme engineering

Enzyme engineering is an innovative approach to exploit a broad range of enzymes with specific properties such as higher reaction rate, better specificity, faster kinetics, less by-products, and more safety nature, which make it the most suitable targets for altering cellular pathways at protein level (Bornscheuer et al. 2012). Three strategies in enzyme engineering have been explored to manipulate and tailor biocatalysts (Lutz 2010, Ali et al. 2020): irrational protein design such as directed evolution; semirational protein design such as sequence-based enzyme redesign, structure-based enzyme redesign, and computational enzyme redesign; rational protein design such as computational *de novo* enzyme design for synthesizing biocatalysts with novel function and recreating existing enzymes.

Irrational protein design is a methodology that involves the creation of extensive libraries of protein variants through random mutations, without the necessity of prior structural or mechanistic insights. This approach is subsequently complemented by screening or selection processes aimed at identifying enhanced phenotypic traits (Bunzel et al. 2021). A notable instance of irrational protein design is the engineering of PETase to enhance the degradation of PET waste (Lu et al. 2022). To improve the hydrolytic activity and thermal stability of PETase, a BHET-OH fluorescence assay was established as a high-throughput screening method to facilitate three rounds of directed evolution. The resulting combinatorial variant M3 (PETase^N246D/Q119R/T88I/D220N/S290P^) showed a 1.6-fold increase in enzymatic activity and a 10.3°C higher T_m_ value compared to those of the wild-type PETase (Shi et al. 2023).

Semirational protein design is based on the understanding of protein sequences, structures, and functions. It uses computational predictions to identify promising target sites or employs site-directed mutagenesis to enhance protein performance (Foo et al. 2012). A good example of semirational protein design is the development of a nonnatural pathway for fluorination reactions. Fluorination, through the introduction of fluorine atoms, can significantly enhance the chemical stability, metabolic stability, and physicochemical properties of molecules, thereby improving their performance and applicability in pharmaceuticals, materials, and other related fields. Enzymatic catalysis facilitates this transformation under mild conditions (Pardo et al. 2022, Haas and Nikel 2023). In this case, *N*-fluoroacetamide (1NF) was utilized as a model substrate to screen over 40 different nonhaem iron enzymes. (*S*)-2-hydroxypropylphosphonate epoxidase (SvHppE) from *Streptomyces viridochromogenes* was obtained, which achieved the formation the desired fluorinated product with a total turnover number (TTN) of 5 and a yield of 3.8%. After confirming the initial activity, directed evolution was applied to progressively improve the catalytic efficiency of SvHppE. A homology model was constructed, and molecular dynamics simulations of the enzyme–substrate complex were conducted. Through tunnel analysis, several key amino acid residues (Y101, Y102, A131, N134, and F181) were identified. Site-saturation mutagenesis at these positions led to the isolation of a triple mutant, SvHppE^N134W/E141D/Y102C^, which produced the target fluorinated product with a TTN of 50, and a yield of 30%. This study established a nonnatural fluorination pathway catalysed by a nonhaem iron enzyme, providing valuable insights for further engineering of such enzymes and presenting a novel strategy for enzymatic fluorination (Zhao et al. 2024).

Rational protein design utilizes computational methodologies to systematically construct biomolecular structures, facilitating de novo development of enzymes with customized catalytic properties and precise optimization of existing biocatalysts through structure–function decoupling strategies (Buller et al. 2025). A prominent instance of rational protein design is de novo creation of luciferases. Given the intrinsic challenges in the design of substrate-binding pockets and catalytic active sites, a deep-learning-based “family-wide hallucination” approach was developed. This innovative approach facilitates the generation of a substantial array of idealized protein structures with diverse pocket geometries, along with the design of corresponding sequences that encode these structures. Employing this strategy, an artificial luciferase was successfully designed and engineered for responding to diphenylterazine (DTZ) and 2-deoxycoelenterazine (h-CTZ). Through optimization and multiple biological assays, LuxSit-i exhibited substrate specificity that surpasses that of natural luciferases. This methodology presents an extensive range of scaffold possibilities for the placement of substrate-binding sites and catalytic residues, thereby providing a robust new pathway for the design of enzyme functions (Yeh et al. 2023).

Enzyme engineering has become a fundamental aspect of synthetic biology and metabolic engineering, facilitating the precise and efficient modification of cellular functions. Although irrational, semirational, and rational design strategies have led to significant advancements in enzyme performance and substrate specificity, several challenges still remain in predicting the effects of mutations, achieving a balance between catalytic activity and stability, and adapting designs for industrial applications (Song et al. 2023, Orsi et al. 2024). In particular, rational approaches frequently prove inadequate due to insufficient structural information or inaccurate computational models, whereas random mutagenesis can generate superior variants but is labor-intensive and lacks mechanistic interpretability. Looking ahead, the integration of machine learning with high-throughput screening and structural modeling is expected to improve predictive capabilities, decrease the experimental burden, and enable the development of more advanced and reliable biocatalysts for industrial applications (Xie and Warshel 2023). To maximize this potential, future strategies need to address challenges related to data quality, model generalizability, and functional validation to enhance the translation of predictions into practical enzymatic improvements.

## Level 2: rewiring pathway

**Figure 3.**
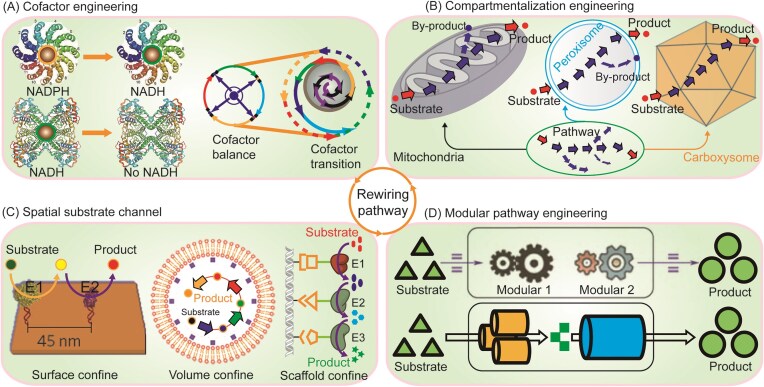
Overview of strategies for rewiring pathway. (A) Cofactor engineering for balancing cofactors supply; (B) compartmentalization engineering for relocating pathway enzyme; (C) spatial substrate channel for building substrate channel; and (D) Modular pathway engineering for coordinating pathway modular.

Expanding on gene-level rewiring, pathway rewiring seeks to integrate multiple optimized parts into coordinated functional pathway. Based on this strategy, a typical approach of synthetic biology for heterologous biosynthesis of target products from renewable resources begins with an efficient pathway with several functional enzymes. However, these synthetic pathways are often assembled from biological components culled from nature, and thus it is basically impossible to function optimally when they simply put together in biological systems. This highlights the limitation of part-level modifications, and emphasizes the necessity of pathway-level rewiring. To address this issue, many attempts in pathway engineering for efficiently increasing production of valuable chemicals have focused on cofactor engineering, compartmentalization engineering, spatial substrate channel, and modular pathway engineering (Fig. 3).

### Cofactor engineering

Cofactor engineering is a useful strategy for renovating enzymes to use the desired cofactor at individual reaction level by protein engineering, and altering NAD(P)H availability at cellular level by coupling cofactor reaction, increasing total NA(P)D^+^ level, overexpressing cofactor-dependent enzymes, and so on. Recent progress in cofactor engineering mainly include two parts (Nielsen et al. 2023): cofactor specificity system for modifying cofactor specificity and creating biorthogonal redox systems; cofactor regeneration system for cofactor interconversion and regeneration.

Cofactor specificity system refers to the molecular mechanisms through which enzymes exhibit high selectivity and dependency toward specific types of cofactors, such as NAD⁺ or NADP⁺, in the course of catalytic reactions (Bachosz et al. 2023). A notable example of cofactor specificity system is the engineering of formate dehydrogenase (Fdh) to alter and expand its intrinsic preference for redox cofactors. In this context, Fdh_+SNO_ from *Starkeya novella* DSM 506 was selected owing to its exceptional chemical stability and strict native specificity for NAD⁺. To reprogram its cofactor preference and enhance its capability for driving NADP⁺-dependent reduction reactions, targeted site-directed mutagenesis was applied to key residues within the cofactor-binding region. Among the engineered variants, Fdh_+SNO_^A198G/D221Q/H379K/I380V^ displayed a significant alteration in specificity, demonstrating enhanced catalytic efficiency toward NADP⁺ compared to its natural electron acceptor NAD⁺. This modification enables more efficient redox balance in NADP⁺-driven biotransformation processes (Partipilo et al. 2023).

Cofactor regeneration system refers to the methodology of incorporating supplementary enzymes or metabolic modules to facilitate the continuous recycling of oxidized or reduced cofactors in a reaction system. This approach aims to maintain catalytic efficiency and enhance economic viability (Sun et al. 2023). An illustrative case of cofactor regeneration is the metabolic engineering of *S. cerevisiae* to optimize acetyl-CoA flux, which subsequently enhances lycopene biosynthesis. The limited availability of cytoplasmic acetyl-CoA has been identified as a bottleneck in downstream metabolic pathways. To remove this bottleneck, acetyl-CoA flux was first redirected toward the mevalonate pathway by optimizing the combination of aldehyde dehydrogenase and acetoacetyl-CoA thiolase. Further, the competing pathway for acetyl-CoA was dynamically downregulated, resulting in a 2-fold increase in lycopene production. Ultimately, the engineered strain W2-A-5 was developed, achieving a lycopene yield of 68 mg/g CW (Su et al. 2022).

Cofactor engineering has shown considerable promise in optimizing intracellular redox balance and broadening the compatibility of enzyme cofactors, which in turn enhances metabolic flux toward desired products. This strategy is highly beneficial for improving yield in redox-demanding biosynthetic pathways. However, several challenges still remain, including inadequate cofactor regeneration, redox imbalances under dynamic conditions, and limited enzyme versatility, thereby limiting its applicability in complex and variable metabolic settings. In the future, cofactor engineering can be significantly improved by incorporating dynamic regulatory elements, allosteric modulation techniques, and systems-level modeling. This approach aims to develop more robust, flexible, and orthogonal redox networks that are specifically designed to meet the intricate demands of biosynthetic processes (Arriaza-Gallardo et al. 2023, Wang et al. 2023b). Such advancements are expected to enable the development of more dynamic and independent redox systems capable of preserving homeostasis under diverse physiological conditions.

### Compartmentalization engineering

Compartmentalization engineering is a direct approach at molecular level to build substrate transmission channel through configuring and controlling key enzymes in organelles to prevent metabolite exchange and circumvent undesirable interactions between heterologous pathways and host cells. Currently, much of the effort in compartmentalization engineering for multiple subcellular locations mainly contains mitochondria, peroxisome, carboxysome, endoplasmic reticulum, Golgi apparatus, vacuole, periplasm, and cell wall (Hammer and Avalos 2017).

Mitochondria, characterized by their distinctive double-membrane architecture, play an important role in cellular metabolism. They primarily facilitate TCA cycle and the oxidative phosphorylation pathway, which are essential for the generation of key metabolites and energy substrates (Zhang et al. 2021). A good instance of compartmentalization engineering is isobutanol production in *S. cerevisiae*. The biosynthetic pathway of isobutanol consists of two parts: the upstream pathway is confined to mitochondria, and the downstream pathway is confined to cytoplasm. When this pathway for isobutanol biosynthesis was partially constructed by overexpressing some enzymes in their natural compartments, isobutanol production was showed only a slight increase, possibly due to the fact that pathway subcompartmentalization created various bottlenecks. Thus, the complete isobutanol pathway was targeted to mitochondria, and thus isobutanol production was increased by 260% compared with that in natural compartments (Avalos et al. 2013).

Peroxisomes are eukaryotic organelles characterized by a single membrane and exhibit permeability to numerous small molecules, generally those with molecular weights below ~500–700 Da. These organelles play a crucial role in a variety of catabolic pathways, including fatty acid β-oxidation, the glyoxylic cycle, and methanol metabolism, in addition to participating in certain biosynthetic processes (Sibirny 2016, Liu et al. 2020a, Grewal et al. 2021). An orthogonal peroxisomal transport system (ScPEX5-oPTS1) was developed in *S. cerevisiae* by utilizing the C-terminal domain of PEX5 from *Arabidopsis thaliana*, in conjunction with its corresponding peroxisomal targeting signal PTS1, as well as a highly efficient targeting peptide oPTS1. This system facilitated the construction of a yeast cell factory for the biosynthesis of α-humulene. In a 5-l fed-batch fermentation, the engineered strain *S. cerevisiae* LC18 produced 17.33 g/l α-humulene with its productivity of 0.22 g/l/h (Zhang et al. 2024a).

The vacuole is separated from the cytoplasm by the vacuole membrane, forming an independent vacuole internal environment, which has many functions such as storage, decomposition, and maintaining cell stability (Yin et al. 2024). Transport limitations of metabolites arise when intricate plant pathways distributed in organelles and tissues are reconstructed in single-cell hosts devoid of the corresponding transport mechanisms. To construct an engineered yeast platform capable of producing tropane alkaloid and scopolamine, the transport proteins-AbPUP1 and AbLP1 located on the surface of the vacuole membrane were first expressed in *S. cerevisiae* CSY1300 to enhance vacuole transport and increase the production of scopolamine and scopolamine. Subsequently, other transport proteins were expressed to further promote the exchange of pathway intermediates between regions and the expansion of NADPH availability, the final strain *S. cerevisiae* CSY1324 produced 480 µg/l hyoscyamine and 172 µg/l scopolamine (Srinivasan and Smolke 2021).

Compartmentalization engineering facilitates the spatial segregation of metabolic pathways, thereby enhancing flux regulation, intermediate channeling, and reducing interference between pathways (Jin et al. 2022, Ye et al. 2022). This approach can also mitigate the toxicity of intermediates and reduce unwanted side reactions. Although significant progress has been made in redirecting essential biosynthetic processes into designated organelles, the current methods are still constrained by the insufficient understanding of compartment-specific transport mechanisms and interorganelle communication, as well as the lack of standardized tools for precise spatial control and predictable system behaviors. Future investigations that integrate synthetic organelle systems, advanced protein targeting technologies, and spatiotemporal modeling are expected to further advance this field, ultimately leading to the development of highly efficient and orthogonal metabolic frameworks in microbial cell factories (Dong et al. 2021).

### Spatial substrate channel

Spatial substrate channel is a common theme throughout biology to optimize cells metabolism in different dimensions from the cell scale across microbial consortia to the protein scale inside cellular enzyme complexes, which plays an important role in improving pathway function and reaction efficiency (Abdallah et al. 2022). Current progress in spatial substrate channel to design and synthesize nanobiological devices is mainly reflected in three aspects (Kuchler et al. 2016, Sweetlove and Fernie 2018): surface- or interface-confined spatial optimization such as biological membranes in living systems and biomembranes *in vitro* systems; volume-confined spatial optimization such as organelles in eukaryotes and a confined environment in prokaryotes; scaffold-confined spatial optimization such as DNA scaffold, RNA scaffold, and protein scaffold.

Surface spatial optimization refers to the restriction of substrate conversion processes to cell membranes, synthetic membranes, or alternative two-dimensional interfaces. This methodology facilitates the localization of enzymes or components of metabolic pathways on the membrane or interface, thereby promoting rapid substrate transport and enabling reactions that are spatially concentrated (Jin et al. 2023). A notable example of surface spatial optimization is the development of liposome-based extracellular artificial organelles on individual living cells. Utilizing a biocompatible approach, multilayered functional liposomes were anchored to the surfaces of living cells to create these artificial organelles. The incorporation of diverse extrinsic functionalities was accomplished without adversely affecting cell viability. The feasibility of enzyme-mediated reactions on these artificial organelles was demonstrated through a cascade reaction involving glucose oxidase and horseradish peroxidase. This methodology presents an advanced chemical tool for the effective modification of cells, facilitating the introduction of new functionalities that enhance or complement the cells’ intrinsic capabilities (Yang et al. 2025a).

Volume spatial optimization entails the confinement of metabolic reaction system within a specified three-dimensional space, facilitating the optimization and regulation of local environmental conditions (Hirschi et al. 2022). A pertinent illustration of volume-confined spatial optimization is the development of a membraneless organelle (MLO) to compartmentalize the ribulose monophosphate (RuMP) pathway in yeasts. By strategically localizing methanol assimilation enzymes within this synthetic phase-separated compartment, the engineered system effectively mitigated the accumulation of cytosolic formaldehyde and reduced its associated cytotoxic effects. Consequently, the optimized strain *S. cerevisiae* ZP03-F4, which incorporates the MLO-targeted RuMP pathway, exhibited an 80% reduction in cytosolic formaldehyde levels compared to that of the control strain *S. cerevisiae* ZP03-F1. This optimization resulted in a 13% enhancement in cell viability, a 261% increase in biomass, and a 95% improvement in methanol consumption, thereby underscoring the substantial advantages of spatial organization at the subcellular level for enhancing pathway efficiency and metabolic resilience (Zhou et al. 2023).

Scaffold-based spatial optimization involves the strategic employment of artificially designed scaffold molecules to spatially arrange multiple enzymes in a structured configuration. This approach aims to improve the synergistic efficiency of sequential reactions in metabolic pathways (Wheeldon et al. 2016). A typical example of scaffold-confined spatial optimization is butyrate production. The biosynthetic pathway of butyrate from acetyl-CoA was constructed and optimized, but the final concentration of butyrate was still low, possibly due to the loss of metabolic intermediates and the catalytic inefficiency of heterologous pathway. To further enhance butyrate production, protein scaffolds were introduced to spatially organize 3-hydroxybutyryl-CoA dehydrogenase (Hbd), 3-hydroxybutyryl-CoA dehydratase (Crt), and trans-enoyl-coenzyme A reductase (Ter). Through the optimization of interaction domains in scaffolds, butyrate production with *E. coli* DSM03 was showed 3-fold higher than that of no scaffold control (Baek et al. 2013).

Spatial substrate channeling presents a flexible framework for enhancing metabolic efficiency by facilitating localized transfer of intermediates, minimizing diffusion losses, and optimizing pathway kinetics. It also offers avenues for improving thermodynamic efficiency and protecting unstable intermediates. Despite significant advancements in utilizing surface interfaces, confined volumes, and molecular scaffolds to develop effective nanobiological systems, challenges still remain, including the need for precise spatial control, dynamic regulation, and scalability (Vanderstraeten and Briers 2020). Moreover, the lack of universally applicable design principles and challenges in real-time validation of substrate flux hinder broader application. The incorporation of programmable scaffolds, responsive materials, and real-time spatial imaging technologies is expected to further propel the design of artificial spatial architectures, thereby unlocking new opportunities for intricate metabolic reprogramming in the field of synthetic biology (Dubey and Tripathi 2021).

### Modular pathway engineering

Modular pathway engineering is a novel approach for strain and pathway optimization by organizing key enzymes into distinct modules and simultaneously varying its expression levels to balance metabolic flux in synthetic pathways. This strategy fulfills three criteria for general application: rationally design but require *a priori* knowledge, host-independent and pathway-independent, only explore a small design space with no need for high-throughput screen. Recently, modular pathway engineering has successfully performed its function through dividing metabolic pathways into three main types (Young et al. 2021): biochemistry-based modular pathway, metabolic branch-based modular pathway, and enzyme turnover rate-based modular pathway.

Biochemistry-based modular pathway engineering categorizes metabolic pathways into distinct functional modules based on the catalytic classifications of enzymes, such as carboxylation, reduction, and transfer. This approach facilitates the independent regulation of enzyme expression and reaction conditions in each module, thereby improving the efficiency of critical biochemical reactions (Garcia and Trinh 2019). A notable example of biochemistry-based modular engineering is the development of a novel CO_2_-fixing pathway known as the reductive tricarboxylic acid branch/4-hydroxybutyryl-CoA/ethylmalonyl-CoA/acetyl-CoA cycle (THETA cycle). This synthetic pathway was strategically designed to enhance the biological capture and conversion of CO_2_ by incorporating two highly efficient natural carboxylation enzymes: crotonyl-CoA carboxylase/reductase and phosphoenolpyruvate carboxylase. These enzymes were organized into two separate carboxylation modules to facilitate CO_2_ fixation with high efficiency. By exploiting the metabolic flexibility of *E. coli*, the THETA cycle was successfully implemented *in vivo*, marking the first demonstration of the feasibility of establishing such a complex CO_2_-fixation pathway in a cellular environment (Luo et al. 2023).

Branch-based modular pathway engineering defines modules according to pivotal branching points in the metabolic network. This approach facilitates the independent optimization of upstream precursor synthesis and downstream product conversion, thereby enhancing the flux distribution toward the desired compounds (Lu et al. 2019). A successful instance of modular pathway engineering is taxol production. The biosynthetic pathway of taxol contains two parts: the upstream isoprenoid pathway for accumulating two building blocks; the heterologous downstream terpenoid pathway for producing taxol precursors. Although this pathway was successfully rewired in *E. coli* and *S. cerevisiae*, its titers were only increased to 10 mg/l, mainly owing to the regional imbalance between downstream and upstream pathway. Thus, the biosynthetic pathway of taxol precursor, taxadiene, was divided into upstream module and downstream module. Then, modular optimization was conducted to increase taxadiene production by fine-tuning promoter strengths and plasmid copy numbers, and its final concentration (1.02 g/l) was showed a 15 000-fold increase compared with the control strain (Ajikumar et al. 2010).

**Figure 4.**
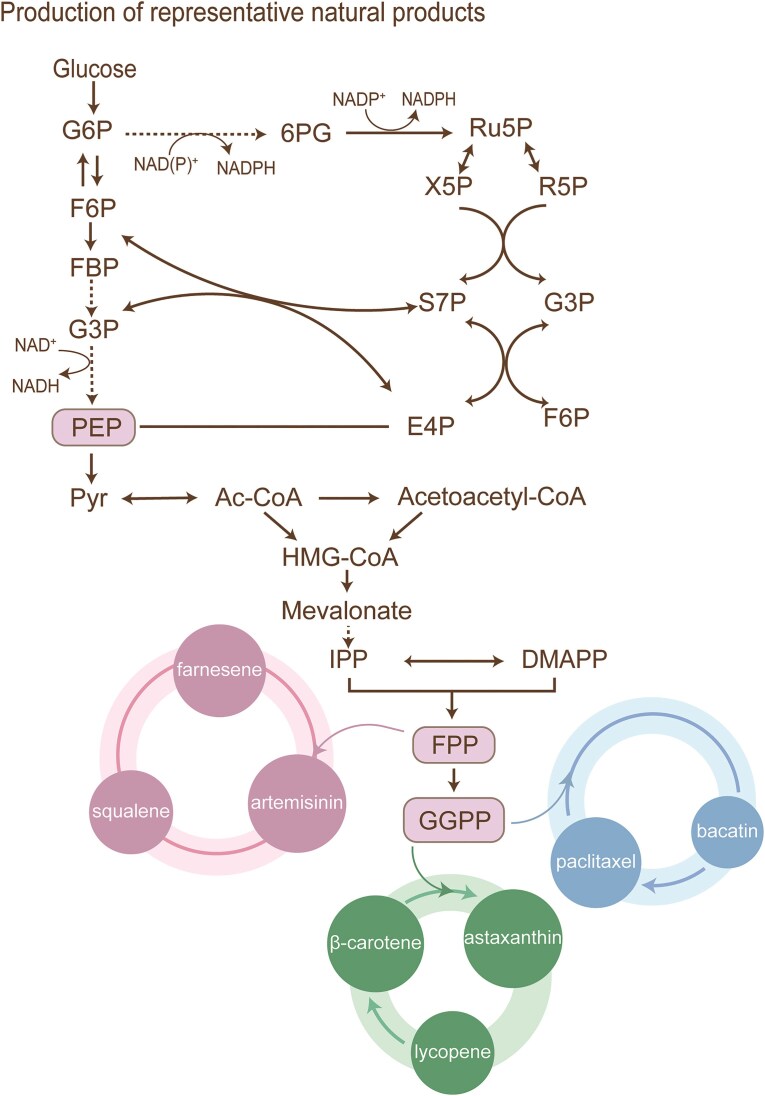
Production of representative natural products. Acetoacetyl-CoA, acetoacetyl coenzyme A; HMG-CoA, 3-hydroxy-3-methyl glutaryl coenzyme A; IPP, isopentenyl pyrophosphate; DMAPP, dimethylallyl pyrophosphate; FPP, farnesyl pyrophosphate; and GGPP, geranylgeranyl pyrophosphate.

Turnover rate-based modular pathway engineering categorizes enzymes into specific modules based on their catalytic efficiency (*k_cat_*/*K_m_*) and utilizes differential expression control of enzymes with high and low catalytic turnover rates to achieve rate matching and sustain dynamic equilibrium throughout the entire biosynthetic pathways. A notable instance of enzyme turnover regulation is the modular control of the mevalonate (MVA) pathway, which was employed to assess its impact on limonene production in *E. coli*. The microbial production of limonene via the MVA pathway imposes a metabolic burden that adversely affects cellular fitness. To mitigate this challenge, a multiinput transcriptional circuit was designed to systematically and independently regulate the expression of the first three enzymes in the MVA pathway-acetyl-CoA acetyltransferase (AtoB), 3-Hydroxy-3-methylglutaryl coenthase A synthase (HMGS), and 3-Hydroxy-3-methylglutaryl CoA reductase (HMGR)-thereby optimizing pathway flux. Through this modular regulation approach, the engineered strain *E. coli* JBEI-6409 was able to produce 76 mg/l limonene, showing a 7-fold enhancement compared to that of the control strain (Shin et al. 2022) (Fig. 4).

Modular pathway engineering offers a systematic and efficient approach to metabolic reconfiguration by breaking down intricate biosynthetic pathways into manageable components that can be optimized independently (Wang et al. 2020b). This modular framework, which can be based on enzymatic functions, metabolic topology, or kinetic parameters, allows for precise regulation of flux distribution, dynamic equilibrium, and pathway efficiency, thereby eliminating the need for exhaustive combinatorial screening. Its inherent standardization also facilitates the reuse and transferability of pathways across different hosts. However, this strategy still faces several limitations, such as the risk of losing global regulatory coordination, issues with module compatibility, and the emergence of unpredictable behaviors upon module integration. In the future, the incorporation of computational modeling, synthetic regulatory elements, and modular assembly toolkits is anticipated to enhance future the development of modular pathway engineering, thereby expediting strain development, enabling pathway scalability, and expanding its applications in the fields of synthetic biology and industrial biotechnology (Wong et al. 2021).

## Level 3: rewiring network

**Figure 5.**
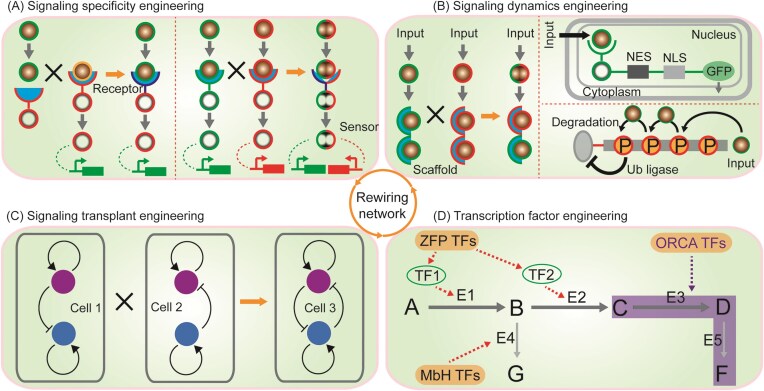
Overview of strategies for rewiring network. (A) Signaling specificity engineering for modifying signaling specificity; (B) signaling dynamics engineering for programming signaling dynamics; (C) signaling transplant engineering for deciphering signal encoding; and (D) Transcription factor engineering for interfering protein translation.

Despite pathway rewiring can be successful at a localized level, the dynamic and interconnected intracellular environments frequently require modulation at the regulatory network level. As the engineered pathways increase in complexity and interact more extensively with host regulatory networks, signal networks allow cells to send, receive, and process information from exogenous interference, such as genetic modification, environmental change, and intercellular communication. Cells can use signal networks to implement diverse functions, such as cell growth, metabolite production, and other complex processes (Green et al. 2014). However, many important signal networks are either incompletely understood or tightly integrated into larger biological systems. This complexity limits the scalability of pathway engineering, making it crucial to consider broader regulatory rewiring to effectively regulate cellular adaptability and responsiveness. To overcome these limitations, synthetic biology offers an alternative bottom-up approach for monitoring, interrogating, and controlling biological networks to improve cell behaviors (Kiel et al. 2010, Nandagopal and Elowitz 2011) through signaling specificity engineering, signaling dynamics engineering, signaling transplant engineering, and transcription factor engineering (Fig. 5).

### Signaling specificity engineering

Signaling specificity engineering is a new field of view for enabling precise spatial and temporal control through modularly engineering cellular signaling receptors and sensors to link various new inputs to a critical cellular response or selectively engaging a single input to modulate intracellular signaling systems. Recent efforts in signaling specificity engineering to control signaling networks largely center on three types of proteins (Yang et al. 2021): extracellular receptor proteins to redirect input sensing and trigger receptor proteolysis; sensor proteins to control cell behavior; and optogenetic proteins to drive a conformational change.

Extracellular receptor proteins are defined as engineered receptor constructs designed to modify the recognition and transduction processes of specific input signals. This process generally entails the integration of a novel input-recognition module with a mechanism for signal release (Zhang et al. 2023). To introduce nonnatural effector control into natural cellular systems, a synthetic regulatory unit was attached noncovalently to glycogen synthase kinase 3 (GSK-3) for enabling it to respond to the nonnatural effector protein lactate dehydrogenase A (LDHA), thereby allowing external modulation of its enzymatic activity. Elevated intracellular LDHA levels under hypoxic conditions led to a substantial increase in GSK-3 activity, whereas downregulation of LDHA expression caused a significant decrease in GSK-3 activity. These findings represent a significant step toward mimicking effector-regulated cellular signal transduction through the use of engineered enzymatic circuits (Suss et al. 2024), showing the potential for engineering nonnatural effector-mediated enzyme activation in living cells.

Sensor proteins are intracellular or membrane-associated proteins that have been modified to react to specific molecular signals or physiological conditions. These proteins are functionally connected to downstream components, including transcriptional regulators and cell cycle control mechanisms (Kinshuk et al. 2024). For example, the chimeric antigen receptors (CAR) domains are able to be recombined with new sensor modules to modularly enhance the specificity of the CAR-T response (Kloss et al. 2013, Wu et al. 2015), and synNotch receptors can be engineered to fully customize both input (target antigen) and output (gene expression) (Morsut et al. 2016). Thus, CAR and synNotch receptors are rewired synergistically to refine the specificity and scope of T cell response (Roybal et al. 2016), which has great potential in treating T cell cancers.

Optogenetic proteins, including LOV, CRY2, and PhyB, are light-sensitive proteins that have been specifically engineered to undergo conformational alterations when exposed to particular wavelengths of light. These conformational changes subsequently initiate various biological processes, including protein activation, oligomerization, dissociation, subcellular relocalization, and the initiation of signaling cascades (Li et al. 2024). A significant illustration of optogenetic proteins is the utilization of light-responsive systems to modulate metabolic reactions in *E. coli* through the dynamic redistribution of enzymes. Photo-activated switches for enzymes was developed to regulate enzymatic activity by manipulating the spatial colocalization of enzymes and their respective substrates. This innovative system is based on scaffold proteins that drive liquid–liquid phase separation to create light-inducible compartments. Upon light illumination, these compartments swiftly recruit enzymes of interest from the cytosol within seconds and can completely release them within 15 min. Furthermore, these compartments enhance the localized concentration of small-molecule substrates, leading to a 2.3-fold increase in luciferin oxidation and a 1.6-fold increase in catechol oxidation. This optogenetic platform provides a means for reversible, tunable, and spatiotemporal control of enzymatic reactions, thereby serving as a valuable tool for exploring the dynamics of protein redistribution and metabolic regulation in *E. coli* (Huang et al. 2022b).

The engineering of signaling specificity represents a groundbreaking approach for the development of synthetic signal transduction pathways characterized by programmable precision. By modularly reconfiguring extracellular receptors, intracellular sensors, and optogenetic regulators, this methodology facilitates the integration of a variety of environmental or cellular signals into precisely controlled response outputs (Spisak and Ostermeier 2020). These engineered signaling circuits not only enhance our comprehension of natural regulatory networks but also create new opportunities for dynamic metabolic regulation, therapeutic interventions, and intelligent cellular systems. However, challenges such as signal leakage, limited orthogonality, and unpredictable context-dependent behaviors still hinder the widespread adoption of this strategy. As synthetic biology toolkits continue to expand and our understanding of signaling cross-talk becomes increasingly refined, signaling specificity engineering is set to become a fundamental component in the design of next-generation bioresponsive platforms and precision control systems (Li and Elowitz 2019).

### Signaling dynamics engineering

Signaling dynamics engineering is repurposed as a tool in synthetic biology for engineering signaling proteins to rewire a posttranslational regulatory network, thus directing cells to perform an appropriate response. Recent progress in signaling dynamics engineering have clarified how to rewire phospho-signaling proteins through three aspects (Gordley et al. 2016): engineering phospho-signaling scaffolds to exert logic gate properties; regulating phospho-regulated linear motifs to create dynamic signaling reporters; and scaling up phospho-circuit design to form information processors.

Phospho-signaling scaffolds are engineered to promote specific protein–protein interactions under carefully defined conditions, thereby facilitating signal transduction behaviors that resemble logical gate functions (Martinez-Val et al. 2021). For example, to augment natural cellular processes such as signal transduction and metabolism, a bistable toggle switch was engineered through reversible protein–protein phosphorylation interactions. This synthetic switch network comprised 11 phosphorylation-based input and output signaling components. The resulting system showed a rapid response to extracellular stimuli occurring within seconds, and maintained long-term bistability throughout cell division. Notably, the network exhibited ultrasensitive switching behavior (Mishra et al. 2021).

Phosphorylation-regulated short linear motifs are modulated to create dynamic signaling reporters capable of real-time monitoring of cellular signaling activities (Moghimianavval 2024). For instance, during the investigation of interactions between calcineurin (CN) and short linear motifs (SLiMs), the unexpected proximities of CN to centrosomal and nuclear pore complex (NPC) proteins were revealed. Subsequently, a conserved role for CN in association with the NPC was confirmed, demonstrating that CN can dephosphorylate NPC proteins in both yeasts and humans, thereby influencing *in vivo* nucleoporin phosphorylation and the nuclear import of reporter genes. These findings provide a framework for the construction of SLiM-based signaling networks and offer significant insights into the overarching architecture of calcineurin-mediated signal transduction in humans (Wigington et al. 2020).

Phospho-circuit design can be achieved by the modular integration of multiple signaling inputs and outputs to create intracellular signaling circuits with computational capabilities for processing stimulus–response interactions (Gordley 2016). A prominent example of this is the engineering of synthetic phosphorylation signaling networks in human cells, which was achieved through the modular assembly of engineered protein domains into reversible enzymatic phosphorylation cycles that form synthetic signaling circuits. These circuits can accommodate diverse network topologies by linking cell-surface receptors for rapid extracellular ligand detection to downstream modules that precisely regulate gene expression. This strategy represents a novel design paradigm for synthetic signal transduction, thereby enabling the accurate emulation and programming of the dynamics of natural signaling networks (Yang et al. 2025b).

Signaling dynamics engineering presents a robust framework for the systematic redesign of regulatory networks, facilitating precise temporal regulation of cellular responses. By utilizing engineered phospho-signaling scaffolds, dynamic linear motif reporters, and modular multiinput phospho-circuits, this methodology not only clarifies the fundamental principles that govern signal processing but also allows for the development of advanced synthetic signaling systems endowed with logical, real-time sensing, and computational capabilities. Despite these advances, it is still challenging for achieving stable temporal resolution, avoiding retroactivity, and ensuring modular compatibility with native pathways. In the future, the convergence of signaling dynamics engineering with other areas of synthetic biology is anticipated to unveil unprecedented opportunities for dynamic metabolic regulation, adaptive cellular behaviors, and programmable therapeutic interventions, thereby broadening the horizons of cellular engineering (Cui et al. 2021, Wu et al. 2024a).

### Signaling transplant engineering

Signaling transplant engineering is a functional innovation in engineering cellular behaviors through transplanting signaling pathways from one organism to another and diverting the outputs of signaling pathways from nonquantifiable native targets to quantifiable reporter genes, thus circumventing the feedback, cross-talk, and induction of dramatic cellular changes. Signaling transplant engineering can use bioinformatics tools to mine natural circuits, design synthetic circuits, or integrate modular circuits that are validated for a high degree of modularity and minimal cross-talk with other cellular components (Hug et al. 2020). These enriched building blocks make it potential to generate new signaling circuits through screening signaling combinations and optimizing signaling pathways. These developments motivate the growing toolbox of regulatory circuits that will be widely used in synthetic biology (Chen et al. 2020c).

The process of mining natural signaling circuits involves the utilization of genomic and transcriptomic databases, along with bioinformatic analyses, to systematically identify and extract signaling pathways from a variety of organisms that demonstrate particular sensing abilities or regulatory dynamics. These naturally occurring circuits can be transferred into heterologous systems and reconstructed to create response modules that are functionally independent from endogenous networks, thereby facilitating the detection and transduction of specific input signals (Şimşek et al. 2023). One successful example, to better understand the physiological plasticity of MAPK pathways, the core mammalian cascade comprising Raf kinase, MAPK kinase, and ERK kinase is ported into yeast, and then Raf kinase is modified to make it directly controllable by b-estradiol. These results indicate that the ultrasensitive response can be independently regulated by altering the relative proportion of kinase components, due to the fact that ultrasensitivity is an inherent feature of signaling cascade. Thus, the core MAPK cascade can act as a tunable amplifier, which is controlled to generate diverse responses (O’Shaughnessy et al. 2011).

The design of synthetic signaling pathways entails the systematic construction of entirely artificial circuits through the modular assembly of sensors, signal-processing components, and output elements. This methodology is predicated on a comprehensive understanding of individual signaling components and underscores the importance of tunable responsiveness, signal amplification, and logic control. Based on this, it is particularly advantageous for engineering cells to detect nonnative signals and to adaptively operate within novel environmental contexts (Xu et al. 2024). A notable illustration of this approach is the enhancement of chemical production through designing genetic circuits to regulate cellular lifespan. A logic gate state machine featuring dual inputs and four outputs was initially developed to modulate the chronological lifespan of cells. The resulting strain *E. coli* BUT-6 increased butyric acid production with its productivity up to 0.414 g/l/h. On the other hand, a bidirectional logic gate state machine was constructed to manage the replicative lifespan of cells. The resulting strain *E. coli* PLH-4 was effectively expanded the accumulation space for PLH (Guo et al. 2020a).

The integration of modular signaling circuits emphasizes the recombination of validated functional modules—such as receptors, adaptors, and transcription factors—into standardized and interoperable units. This strategy prioritizes the standardization of interfaces and the insulation of signals between modules, thereby ensuring high levels of controllability, predictability, and minimal cross-talk in host cells. It facilitates the scalable design and rapid prototyping of synthetic signaling systems (Kim and Simmel 2022). A prominent example of gene circuit modularization is the development of the Multiplex Yeast Toolkit (MYT), which significantly enhances the flexibility and efficiency of genetic engineering in *S. cerevisiae*. By incorporating integration vectors, selectable markers, and CRISPR-Cas9 tools, MYT enabled precise and multiplexed genetic modifications. Further, three orthogonal inducible promoter systems in MYT allowed for finely tuning gene expression, providing a powerful platform for the construction and regulation of complex genetic circuits in yeasts (Shaw et al. 2023).

Signaling transplant engineering presents a flexible methodology for the decoupling and reconfiguration of cellular signaling in a programmable fashion. This technique facilitates the transplantation of natural circuits, the construction of synthetic pathways, and the modularization of functional units, thereby allowing for precise regulation of gene expression and cellular behavior while minimizing cross-talk. Nonetheless, the complexity of network rewiring, the limited principle of quantitative design, and the potential interference of endogenous signaling remain critical challenges that need to be addressed. As the available toolkit continues to grow and the underlying design principles evolve, signaling transplant engineering is anticipated to emerge as a fundamental component in the development of intelligent, responsive synthetic systems that are capable of dynamic decision-making and adaptive biosynthesis.

### Transcription factor engineering

Transcription factor engineering is a valuable alternative to control multiple steps in a particular metabolic pathway for overcoming the bottlenecks of metabolic flux involving multiple enzymatic steps, or deploying pathway genes in specific organelles, organs, and cell types where they normally do not express. Recently, transcription factor engineering has attracted wide interests in three types (Deng et al. 2022): zinc-finger protein transcription factors to fine-tune cascade catalysis; MYB and bHLH transcription factors to match metabolic complexity; and octadecanoid-responsive catharanthus AP2/ERF-domain (ORCA) proteins to control the biosynthetic pathways of secondary metabolites as well as other examples, such as ATF3 (Liu et al. 2024a), MexT (Wang et al. 2024c), and NAC (Bi et al. 2023).

Zinc finger proteins represent a distinct category of transcription factors characterized by their stable DNA-binding domains, which can be modularly engineered to recognize specific DNA sequences. These proteins can be customized into programmable regulatory elements that facilitate the precise modulation of gene expression across multiple pathways, thereby enabling hierarchical and cascade control of intricate metabolic processes (Hamed et al. 2021). For instance, the accumulation of the microtubule-associated protein tau is associated with extensive neurodegeneration. To mitigate tau accumulation and develop effective therapeutic strategies for Alzheimer’s disease and related tauopathies, an innovative approach was devised to specifically and durably suppress endogenous tau expression. This was achieved through the delivery of adeno-associated viruses (AAVs) that encode arrays of zinc finger proteins (ZFPs), which were designed to target a specific short genomic region of the *MAPT* gene. ZFPs were fused to the KRAB repression domain of the human KOX1 transcription factor, facilitating the transcriptional repression of *MAPT*. Pharmacological assessments indicated that a single administration of AAV was adequate to suppress all isoforms of tau mRNA and protein in the brain by 50%–80% for 1 year (Wegmann et al. 2025).

MYB and bHLH transcription factors, which are prevalent in plants, exhibit significant regulatory potential over the biosynthesis of various secondary metabolites and frequently operate in concert as heteromeric complexes. Their capacity to coordinate the expression of multiple metabolic genes in response to environmental stimuli renders them particularly adept at managing structurally complex and highly branched metabolic networks (Ma and Constabel 2019). A notable instance of MYB transcription factors orchestrating complex metabolic reprogramming is their involvement in regulating pancreatic cancer cell metabolism under hypoxic stress. Under low-oxygen environments, MYB enhances cell survival by instigating a metabolic shift that facilitates adaptation to the hypoxic microenvironment. It activates a transcriptional program that includes glycolytic and glutaminolytic genes, thereby promoting glucose uptake and glutamine utilization to sustain energy homeostasis and redox balance. This underscores the capacity of MYB to integrate and address diverse metabolic requirements in pathophysiological contexts, highlighting its potential role as a master regulator of cellular metabolism (Anand et al. 2023).

ORCA proteins, which are pivotal regulators of secondary metabolism in plants—especially in the biosynthesis of alkaloids—can be harnessed to robustly activate the expression of biosynthetic pathways specific to plant-derived natural products (Yamada and Sato 2021). For example, vinblastine biosynthesis can be started from strictosidine via the condensation of vindoline and catharanthine, but this biosynthesis pathway for dimeric alkaloids is tightly induced by internal signals such as jasmonate. As jasmonate-responsive transcription factors, ORCA2/ORCA3 overexpression results in the upregulation of multiple genes in alkaloids biosynthesis pathway, such as cytochrome P450 reductase, tryptophan decarboxylase, strictosidine synthase, strictosidine β-d-glucosidase, and desacetoxyvindoline 4-hydroxylase. This upregulation selectively activates the biosynthetic pathway of vinblastine, leading to a large increase in its formation (van der Fits and Memelink 2000).

Transcription factor engineering represents a robust approach for the precise modulation of multistep metabolic pathways and the facilitation of context-dependent gene expression. This strategy holds promise for customizable, context-dependent regulation, but it frequently faces constraints such as the limited specificity, unintended interactions, and challenges in achieving precise dosage control within complex regulatory networks. Anticipated advancements in modular design and synthetic regulatory networks are likely to provide precision, dynamism, and scalability in the regulation of cellular metabolism. This progress is expected to facilitate the development of increasingly complex applications in the field of metabolic engineering (He et al. 2023, Kayani et al. 2024).

## Level 4: rewiring genome

**Figure 6.**
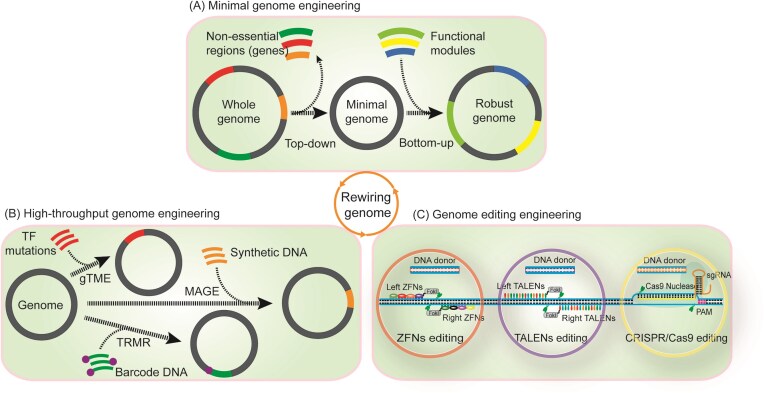
Overview of strategies for rewiring genome. (A) Minimal genome engineering for architecting genome; (B) high-throughput genome engineering for mapping genome; and (C) genome editing engineering for editing genome.

Although rewired networks can influence cellular behaviors, their performance is constrained by the genomic environment in which they operate. To support network-level interventions with comprehensive system-wide effects, metabolic engineering has expanded from a focus on simple designs requiring a small number of genetic modifications to complex designs using genome-scale engineering technologies to understand and engineer microbial genomes for efficient biosynthesis of fuels, chemicals, and drugs. Usually, traditional approaches are labor-intensive, time-consuming, and difficult for rational genome modifications, strain analysis, and strain characterization. Therefore, to fully realize the potential of network rewiring, genome-scale interventions become necessary to establish a stable and high-throughput platform for controlling system-level functions. Recent progress in genome-scale engineering have overcome these challenges, and thus greatly expand our ability to reprogram biological systems by minimal genome engineering, high-throughput genome engineering, and genome editing engineering (Fig. 6).

### Minimal genome engineering

Minimal genome engineering is based on removing nonessential parts of the genome, only leaving the parts required for an organism to replicate in a given environment. Currently, two approaches for the construction of minimal genomes have been proposed (Rees-Garbutt et al. 2021): top-down approach to further simplify existing cells by removing nonessential genes; bottom-up approach to create an artificial cell by synthesizing essential components.

The top-down approach streamlines the understanding of cellular functions by methodically deleting nonessential genes from a well-characterized organism, ultimately preserving the core genetic components necessary for minimal viability (Hutchison et al. 2016). A pertinent illustration of this approach is the genome-wide analysis of interactions between essential and nonessential genes in *Streptococcus pneumoniae* using gene editing technologies and omics-based methodologies. The CRISPRi-TnSeq technique, which combines CRISPR interference-mediated repression of essential genes with transposon sequencing (TnSeq) of nonessential gene knockouts, facilitated a comprehensive examination of genetic interactions. In total, transposon mutant libraries were developed across 13 CRISPRi backgrounds, enabling the screening of ~24 000 gene pairs. This investigation identified 1334 genetic interactions, which included 754 negative and 580 positive interactions. Network analysis indicated that 17 nonessential genes displayed pleiotropic interactions with half of the tested essential genes. Experimental validation demonstrated that a subset of seven genes could mitigate cellular disturbances. Consequently, CRISPRi-TnSeq platform was proved to be an effective tool for revealing synthetic and suppressive relationships between functionally related and distinct pathways, thereby uncovering latent redundancies that compensate for the loss of essential functions, particularly those associated with cell wall synthesis, integrity, and division (Jana et al. 2024).

Conversely, the bottom-up strategy in synthetic biology seeks to reconstruct artificial cellular systems capable of performing fundamental life processes through the chemical reconstitution of essential genomic modules. This approach emphasizes modular design and the analysis of minimal functional units, providing a programmable framework to investigate the controllable assembly and emergent behaviors of living systems (Marquez-Zavala and Utrilla 2023). A notable example of this strategy is the construction of a gene-programmed artificial cell chip that exhibits collective gene expression dynamics. On a microfluidic chip measuring 5 × 5 mm², an array of 1024 interconnected synthetic cell units was fabricated using photolithographic techniques. Each unit was equipped with immobilized DNA brushes to facilitate *in situ* programming of gene circuits and control of steady-state expression. By arranging the units into a square lattice, a discrete reaction-diffusion network was established. The integration of a nonlinear oscillator genetic circuit, consisting of mutual repressor modules, successfully resulted in collective oscillation and the propagation of traveling waves across the array. The microscopic dimensions of the unit chambers and microcapillaries influenced the effective diffusion and coupling strength within the lattice, which subsequently governed the macroscopic synchronization dynamics. Strongly coupled oscillators exhibited rapid and continuous two-dimensional wavefronts emanating from the boundaries, leading to smooth oscillator phase gradients and extensive spatial correlations. This research paves the way for a new class of nonequilibrium, genetically programmed synthetic multicellular systems in two dimensions, where the dissipative chemical energy of protein synthesis drives the emergence of large-scale spatiotemporal patterns (Ricouvier et al. 2024).

Minimal genome engineering presents a potent avenue for dissecting life at its most fundamental level through the reduction of native genomes or the construction of artificial systems from the ground up. This strategy facilitates the development of efficient and well-defined cellular platforms, leading to reduced regulatory complexity and improved predictability. However, challenges remain, such as the potential trade-offs between simplicity and flexibility, as well as the possibility of excluding nonessential yet advantageous functions. With the progress of genome editing, synthetic biology, and systems modeling, future endeavors are likely to concentrate on the integration of these methodologies to design minimal yet robust chassis for tailored cellular functions and foundational biological research (Rees-Garbutt et al. 2020, Liu et al. 2022).

### High-throughput genome engineering

High-throughput genome engineering is an art for multiply modifying endogenous genes and regulatory elements in cellular network to overcome limitations of biological systems to gain the desired functions (Huang et al. 2022a, Li et al. 2022). Recently, powerful methods for high-throughput genome engineering have been developed, such as global transcriptional machinery engineering (gTME) (Celińska and Zhou 2025), multiplex automated genome engineering (MAGE) (Wannier et al. 2021), coselection MAGE (CoS-MAGE) (Wang et al. 2024a), DNA replication forks editing-based eukaryotic MAGE (eMAGE) (Barbieri et al. 2017), trackable multiplex recombineering (TRMR) (Warner et al. 2010), conjugative assembly genome engineering (CAGE) (Güell 2020), RNAi-assisted genome evolution (RAGE) (Chen et al. 2020b), serine recombinase-assisted genome engineering (SAGE) (Elmore et al. 2023), and recombineering-based Exponential Replacement (REXER) (Antoine 2025).

gTME refers to a strategic approach for introducing mutations, rearrangements, or directed evolution into critical transcription factors or fundamental components of the transcriptional machinery, including RNA polymerase subunits and general transcription factors. This methodology facilitates large-scale reprogramming of the cellular transcriptome across the entire genome. Consequently, it enables the screening and identification of strains exhibiting desirable phenotypes, such as improved production capability or increased stress tolerance (Velazquez Sanchez et al. 2023, Antoine 2025). A classic instance of gTME is l-tyrosine production. As a branch of the shikimate pathway, the biosynthetic pathway of l-tyrosine has been engineered and optimized, but it is difficult to achieve the high efficiency of l-tyrosine production, possibly due to the unpredictable disconnects between genotypes and phenotypes in biological systems. Thus, to overcome these disconnects, gTME was used for constructing the libraries of *E. coli* P2 by introducing mutations into two global regulators: the RNA polymerase α subunit (*rpoA*) and the sigma factor σ^70^ (*rpoD*). Then, these mutations were introduced into host strain harboring the *aroGD146N-CM/PDH M53I/A354V* operon. After that, three mutants were isolated, and the highest concentration of l-tyrosine (13.8 g/l) showed a 114% increase compared with that of the original strain *E. coli* P2 (Santos et al. 2012). SAGE refers to a modular and efficient platform that utilizes serine recombinases to achieve site-specific, iterative integration of exogenous DNA into the genome without the need for plasmid maintenance. By enabling precise, marker-recyclable insertion of multiple genetic elements across successive rounds, this toolkit supports the stable and scalable construction of engineered strains in both model and nonmodel microorganisms, thereby accelerating applications ranging from metabolic pathway optimization to the development of robust industrial production strains (Elmore 2023 ). A representative example is the PKS-based retrobiosynthesis of δ-valerolactam (VL) and three α-substituted VL analogues in *Pseudomonas putida*. To prevent the degradation of the target products, three loci associated with lactam degradation (oplBA, PP_5182, davAB) were deleted, generating the LP strain specifically designed for heterologous lactam biosynthesis. Using SAGE, three crucial genetic modules—β-amino acid loading, chimeric PKS, and extender unit biosynthesis—were sequentially integrated into different genomic sites, facilitating the stable construction of a complete C5-lactam biosynthetic pathway and the successful production of α-ethylvalerolactam. Through the combination of AT-domain exchange, codon optimization, and metabolic pathway engineering, the researchers achieved the efficient production of enantiopure VL analogues from renewable carbon sources. Subsequently, these lactam derivatives were polymerized into polyamides or *N*-acryloyl derivatives, demonstrating their potential as bio-based monomers for advanced material applications (Lee 2025).

REXER represents a genome rewriting technique that utilizes recombineering to facilitate the stepwise and exponential replacement of multiple genomic segments with designed sequences throughout an entire chromosome (James et al. 2025). For instance, in *E. coli*, the REXER platform allows for large-scale genomic replacement of up to 100 kb through bacterial artificial chromosome donors. This procedure involves the electroporation-mediated introduction of donor DNA, followed by Cas9-mediated linearization and λ-Red recombinase-driven homologous recombination (Wang et al. 2016, Fredens et al. 2019). Further, the GENESIS system extends this framework by permitting iterative REXER-driven replacement of contiguous genomic regions, thereby facilitating modular genome engineering. Conversely, the CONEXER platform leverages high-efficiency conjugative transfer to optimize the integration workflow, achieving seamless DNA insertion in a single day (Zürcher et al. 2023). Additionally, the targeted deletion of 20 endogenous host factors significantly reduces off-target recombination between the native genome and synthetic constructs, thereby improving integration fidelity.

High-throughput genome engineering has emerged as a potent strategy for reconfiguring cellular networks and addressing functional limitations by enabling multiplexed genetic modifications. Its primary advantages are the acceleration of strain development and functional screening on a large scale. Nevertheless, this approach may encounter technical challenges, such as the limited efficiency of gene editing in specific organisms, challenges in preserving genomic stability, and the inadvertent accumulation of unintended interactions. Future endeavors are anticipated to emphasize enhanced precision, increased scalability, and seamless integration with computational tools for design optimization (Chen et al. 2020a). As editing fidelity continues to improve and automation becomes more readily available, this approach is expected to expand its utility in the construction of complex phenotypes, thereby supporting applications ranging from metabolic rewiring to synthetic genome (Lian et al. 2019, Rothstein et al. 2020).

### Genome editing engineering

Genome editing engineering is a powerful method to enable efficient and precise genetic modifications in a diverse range of cell types and organisms by creating targeted DNA double-strand breaks to stimulate cellular DNA repair mechanisms. According to editing mechanisms, three different methods have been explored to edit genome efficiently (McCutcheon et al. 2024, Pacesa et al. 2024), including zinc-finger nucleases (ZFNs) editing, transcription activator-like effector nucleases (TALENs) editing, and clustered regularly interspaced short palindromic repeats (CRISPR) editing.

ZFNs are artificial restriction enzymes composed of a zinc finger DNA-binding domain fused to a FokI endonuclease domain. The zinc finger motifs are responsible for the recognition and binding of specific DNA sequences, while the FokI domain dimerizes to facilitate targeted double-strand breaks (DSBs) in DNA. By creating various zinc finger modules, ZFNs can be tailored to cleave nearly any genomic location, thereby activating the cellular DNA repair mechanisms necessary for gene editing (Li et al. 2020). One representative example involves the development of functional ZF-ND1 pairs and the enhancement of ZFN genome editing efficiency. A modular assembly system was employed to construct 6-finger ZF-ND1s, and their performance was optimized using AlphaFold, Coot, and Rosetta. Among the 10 ZFN variants tested, two variants exhibited functional genome editing activity. Subsequently, structure-guided engineering with AlphaFold, Coot, or Rosetta was conducted, leading to a 5% improvement in editing efficiency. This result demonstrated the effectiveness of structure-based modeling for optimizing ZFN functionality (Katayama et al. 2024).

TALENs, similar to ZFNs, are chimeric enzymes comprising a DNA-binding domain and a FokI nuclease domain. The DNA-binding region is derived from TALE proteins of plant pathogenic bacteria, with each TALE repeat recognizing a single specific nucleotide, offering a highly modular and customizable targeting capability. TALENs bind to sequences flanking the target site, enabling FokI dimerization and the induction of DSBs for efficient genome editing (Sakuma and Yamamoto 2023, Morimoto et al. 2024). A representative example of TALEN-based genome editing is the development of a highly efficient and heritable gene disruption platform in *Xenopus embryos*. By optimizing the TALEN system, a highly efficient and heritable targeted gene editing platform was established in *Xenopus laevis* and *Xenopus tropicalis*. TALEN arrays were constructed using the Golden Gate assembly strategy, and heterodimeric FokI variants (ELD/KKR) were introduced to enhance cleavage specificity and reduce cytotoxicity. A total of eight TALEN pairs targeting different genes were systematically designed based on optimized targeting rules. Functional assays showed that microinjection of TALEN mRNA into embryos induced somatic mutation rates as high as 95.7%, with biallelic disruption efficiencies reaching 80%–90%. BLAST analysis and sequencing further confirmed a low risk of off-target effects (Lei et al. 2012).

CRISPR represent the most widely used genome editing system to date. The core components of the CRISPR-Cas system include a Cas nuclease and a guide RNA (gRNA). gRNA directs Cas protein to the target DNA sequence through base pairing, enabling site-specific DNA cleavage. Compared to ZFNs and TALENs, the CRISPR system offers simpler design, higher scalability, greater throughput, and higher editing efficiency, and has been broadly applied across organisms ranging from prokaryotes to mammals (Anzalone et al. 2020, Doudna 2020, Makarova et al. 2020, Kaminski et al. 2021). A prime example of genome editing engineering is β-carotene production. The biosynthetic pathway for β-carotene production mainly contains five modules: glycolysis module, pentose phosphate module, 2-C-methylderythritol-4-phosphate module, mevalonate module, and β-carotene synthesis module. Due to the complexity and multiple metabolic pathways, taking genetic modification is a low-efficiency and time-consuming procedure. Thus, CRISPR/Cas9 editing was adopted for various genomic modifications, such as gene insertion, deletion, and replacement, and these modifications were completed within 2 days per cycle with nearly 100% editing efficiency. Based on this, more than 100 genetic variants were formed, and the best mutation *E. coli* ZF237T was able to produce 2.0 g/l β-carotene after combinatorial optimization (Li et al. 2015).

Genome editing engineering has revolutionized genetic manipulation by enabling precise, efficient, and programmable modification across diverse organisms. It offers high precise and adaptable gene editing capabilities without the incorporation of exogenous components. However, challenges persist in the form of off-target impacts, context-specific variability, and limitations in delivery methods, thereby hindering widespread application. Future developments will likely focus on enhancing editing specificity, minimizing off-target effects, and integrating editing tools with real-time cellular monitoring. The convergence of genome editing with machine learning, protein design, and synthetic biology is expected to yield next-generation platforms with greater control over spatiotemporal gene regulation, supporting applications from therapeutic correction to whole-genome rewriting (McCarty et al. 2020, Chen et al. 2022, Duan et al. 2024, Eggers et al. 2024, Zhang et al. 2024c).

## Level 5: rewiring cell

**Figure 7.**
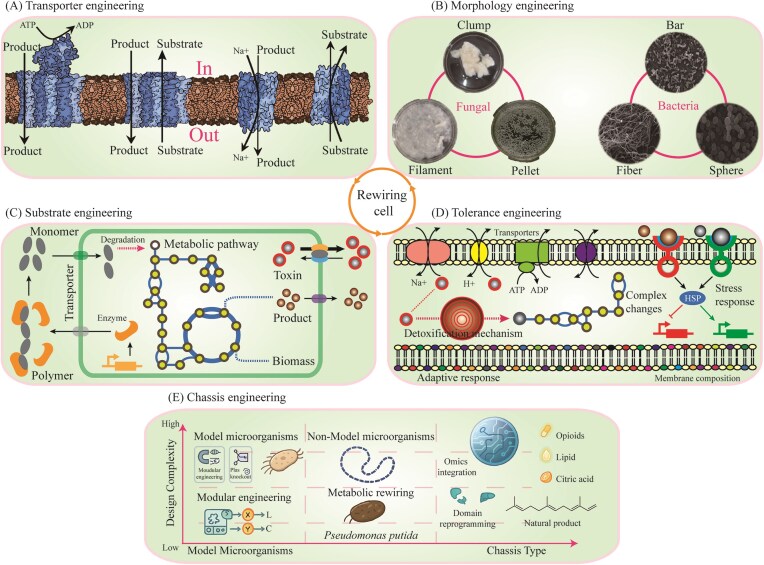
Overview of strategies for rewiring cell. (A) Transporter engineering for improving substrate uptake and product export; (B) morphology engineering for altering cell volume; (C) substrate engineering for engineering substrate utilization; (D) tolerance engineering for elevating stress tolerance; and (E) chassis engineering for enhancing genetic stability, metabolic activity, environmental adaptability, and capacity for product biosynthesis;

**Figure 8.**
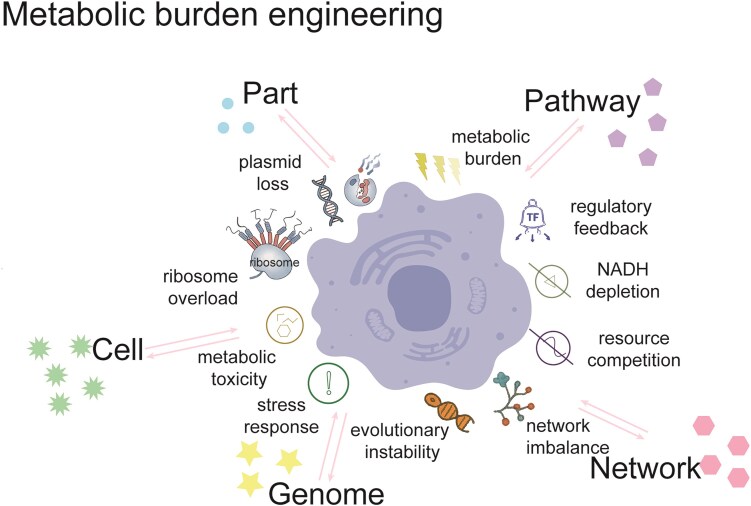
Metabolic burden engineering at part, pathway, network, genome, and cell levels. Metabolic burden spreads through various levels including parts, pathways, networks, genomes, and cells due to factors like plasmid loss, ribosome overload, NADH depletion, and regulatory imbalances. These disruptions do not operate in a single layer but interconnect dynamically.

The cumulative impact of engineering layers-from gene to genome level-manifests within the cellular milieu. To achieve reliable and scalable production, the comprehensive integration of cell physiology is imperative. At cell level, metabolic burden is an imbalance in proportioning the resource of a host cell such as energy molecules or carbon building blocks, which has a direct impact on the productivity of industrial strains (Shabestary et al. 2024). Thus, it is extremely important to explicitly consider metabolic burden at any stage of strain development. The traditional methods rely heavily on random mutations or adaptive evolution to reduce metabolic burden in the process of biosynthesis of desired products. This underscores the limitation of focusing solely on genetic or regulatory interventions, and emphasizes the necessity for integrated rewiring at cell level to alleviate systemic limitations. Recent efforts in synthetic biology have already offered more elegant strategies to reduce metabolic burden and balance cell bioproduction by transporter engineering, morphology engineering, substrate engineering, tolerance engineering, and chassis engineering (Fig. 7 and Fig. 8).

### Transporter engineering

Transporter engineering is an effective metabolic engineering method to improve strain tolerance and construct high-yield production strains through modulating the assembly of pumps and the expression of pump regulators. Generally, transporter engineering mainly centers on two categories (Ahmed et al. 2021): exporters should be enhanced to minimize the feedback inhibition and growth toxicity of intracellular chemicals (Wu et al. 2025); importers can be decreased to prevent the entrance of extracellular products, or increased to enhance the absorption of extracellular nutrients (van der Hoek and Borodina 2020).

To mitigate the feedback inhibition of metabolic enzymes and the potential cytotoxic effects resulting from the intracellular accumulation of metabolites, it is beneficial to upregulate the expression of exporter proteins. This enhancement facilitates the efficient excretion of intracellular compounds, thereby sustaining metabolic flux and maintaining the stability of cell growth (Wu et al. 2024b). A shining instance of transporter engineering is limonene production. The biosynthetic pathway of limonene is originated from the mevalonate pathway in eukaryotes or the DXP pathway in prokaryotes. When limonene synthase from *Mentha spicata* and farnesyl diphosphate synthase from *Abies grandis* were overexpressed in *E. coli* BL21 (DE3), only 2.7 g/l limonene was obtained (Willrodt et al. 2014), possibly due to its toxicity and volatility. To address these bottlenecks, efflux pumps from bacterial genomes were screened using bioinformatics, and 43 heterologous pumps were overexpressed in the limonene-producing *E. coli* strain, respectively. When an efflux pump from *Alcanivorax borkumensis* was overexpressed, limonene yield was resulted in a 1.6-fold improvement compared with that of no pump (Dunlop et al. 2011).

Conversely, the expression of importer proteins may be downregulated to inhibit the uptake of extracellular products or toxic substances, thereby alleviating metabolic burden. Alternatively, these proteins can be upregulated to enhance the absorption of nutrients or substrates, which in turn increases metabolic flux and promotes the biosynthesis of desired products (Davies et al. 2024). A notable instance involves the metabolic engineering of *E. coli* to function as an efficient producer for glutarate production. In this process, lysine degradation pathway was harnessed, and further modular analysis indicated that the transport of putrescine posed a rate-limiting step due to the accumulation of putrescine. To address this limitation, the conversion of putrescine to 4-aminovalerate was successfully promoted by the overexpression of the *potE* gene to enhance its reuptake, achieving nearly a 10-fold increase in production efficiency compared to that of the control strain (Li et al. 2019).

Transporter engineering remains a critical component in the optimization of microbial cell factories, particularly in the modulation of metabolite exchange across cellular membranes. It allows for precise regulation of nutrient absorption and product export, crucial for relieving intracellular blockages and avoiding product inhibition. However, the intricate functionality and situational variability of many transporters particularly those with unidentified substrate specificity can pose challenges in their characterization and implementation. Future research endeavors are expected to focus on the rational design and dynamic regulation of transport systems to achieve real-time equilibrium of intracellular metabolite concentrations (Sengupta et al. 2022, Zhou and Lu 2022). The integration of transporter engineering with systems biology and machine learning methodologies may facilitate predictive control over nutrient assimilation and product excretion, thereby improving the robustness of microbial strains and enhancing production efficiency (Oreb 2020).

### Morphology engineering

Morphology engineering offers a new technological framework to explore, discover, and engineer the multiple correlation between morphological development and morphological control by incorporating the concepts and techniques of biochemical engineering and metabolic engineering at macromorphogenesis level. Morphology engineering can be applied to create and optimize cell factories though engineering the relationship between cell morphology and product formation. Recent progress in morphology engineering concentrates mainly on two aspects: fungal morphology can be controlled to affect colony morphology and change cell wall (Gramelsberger 2020); bacteria morphology can be regulated to alter particle interaction, pellet formation, and pellet aggregation, and change cell shape into mini-cell, filamentary cell, and sphere cell (Guo et al. 2020b).

Fungal morphology particularly filamentous species can be regulated to affect colony architecture and alter the composition or structure of cell wall. By adjusting growth patterns, this approach can facilitate mass transfer, oxygen diffusion, and metabolite secretion, thereby optimizing production efficiency in fungal cell factories (Böl et al. 2021). An exemplary demonstration of this approach is the strategic design of the morphology of *Trichoderma reesei* to facilitate the efficient biosynthesis of l-malic acid (MA). The fungal morphology of *T. reesei* OEtrmae1-trpyc-trhxt2-Δgul1was engineered by deleting the *gul1* gene to reduce the formation of smaller mycelial clusters and the viscosity of broth, thereby optimizing its production performance in bioreactors and increasing MA production. In a 5-l bioreactor, the engineered strain *T. reesei* OEtrmae1-trpyc-trhxt2-Δgul1 produced 100.89 g/l MA, which was increased by 11.65% compared with that of the parental strain (90.36 g/l) (Zhao et al. 2021, Chen et al. 2023).

Bacterial morphology regulation refers to the manipulation of bacterial cell shapes to affect intercellular particle interactions, pellet formation, and aggregation behaviors. This process involves reshaping cells into specific forms, such as minicells, filamentous cells, or spherical cells, which can be utilized to enhance the stability of bioprocess, facilitate downstream separation, or increase productivity (Huo et al. 2020). An excellent example of morphology engineering is polyhydroxybutyrate (PHB) production. As inclusion body, PHB accumulation was achieved in many bacteria through combining PHA biosynthesis and succinate degradation, but this accumulation was limited due to the amount of PHB granules and the quantity of PHB in each cell. To further improve PHB production, the cell size of *E. coli* JM109SG was enlarged by overexpressing actin-like protein (*mreB*), 6-hydroxymethyl-7,8-dihydropterin pyrophosphokinase (*folK*), SOS cell division inhibitor (*sulA*), p*haCAB operon, Cat2, 4HBd, SucD*, and *IspH*. Finally, 87% w/w PHB was obtained in the engineered *E. coli* JM109SG (ΔmreB/pTK-mreB-P*_BAD_*:sulA/pBHR68) (Jiang et al. 2015). These results indicated that morphology engineering has significant potential for biosynthesis of inclusion bodies, such as PHA, proteins, and carboxysomes.

Morphology engineering is increasingly recognized as a significant approach to connect cellular architecture with biosynthetic efficacy. By altering cell shape and compartmentalization, this strategy can improve precursor channeling, enhance enzyme retention, and facilitate spatial arrangement of biosynthetic modules. However, excessive or improperly regulated morphological changes may disrupt cellular homeostasis, division, or viability, particularly in large-scale industrial settings. Future advancements are expected to focus on the amalgamation of morphological regulation with metabolic and transcriptional control, facilitating the precise adjustment of cellular shape, size, and organization for customized bioproduction. The utilization of machine learning-assisted morphometric analysis and synthetic circuit-guided morphogenesis may further enhance the design possibilities in the realm of morphology engineering. By integrating structural manipulation with dynamic metabolic responses, this methodology presents considerable potential for achieving enhanced efficiency, particularly in the synthesis of structurally related biomaterials and inclusion bodies (Mills et al. 2022, Lu et al. 2024).

### Substrate engineering

Substrate engineering is a new indicator in biosynthetic chemistry to achieve sustainable production, which has great potential in engineering microbial cell factories to convert renewable feedstocks, bio-based raw materials, or toxic industrial wastes into added-value products (Nurwono et al. 2023). Substrate engineering can be adopted for modifying the enzymatic machinery in microorganisms to cope with inexpensive carbon and nitrogen sources. These functional modifications generally focus on five aspects (Ledesma-Amaro and Nicaud 2016): (i) engineering extracellular mechanisms to broke down substrates into their subunits by enhancing enzyme secretion out of cell or enzyme display at cell surface; (ii) engineering transport mechanisms to delivery the desired substrate subunits by expressing or selecting specific transporters; (iii) engineering degradation pathways to catabolize substrate subunits by expressing the absent or inefficient enzymes in microbial host; (iv) engineering central and specific metabolic pathways to improve cell growth in the desired substrates by adjusting bottlenecks and regulatory proteins in different pathways for substrate consumption; and (v) engineering cell tolerance to toxic compounds to improve the yield and productivity of target products by preventing it from entering cells, degrading it rapidly inside, inactivating it via conjugation, or pumping it out of the cell. A significant instance of substrate engineering is production of low-cost biofuels. To reduce the raw material cost, α-amylase from rice and glucoamylase from *Aspergillus niger* were expressed in *Yarrowia lipolytica*, and the engineered strain could grow on both soluble starch and raw starch. Further, lipid production was improved by adding a second copy of each amylolytic enzyme with both kinds of starches as substrate (Ledesma-Amaro et al. 2015). These results opened up a new thought for biofuel production from starch in the not-too-distant future.

Substrate engineering increases substrate availability for metabolic activity and augments the biosynthetic potential of host organisms (McDonald et al. 2022). Conversely, degradation pathway engineering focuses on the introduction or optimization of intracellular enzymatic pathways that facilitate the catabolism of substrate subunits, which may otherwise accumulate or remain underutilized due to the inefficacy or absence of native enzymes. A notable example is the alleviation of substrate inhibition in lycopene cyclase, thereby promoting high-efficiency carotenoid biosynthesis. The substrate inhibition of lycopene cyclase (LC) is a key factor for affecting the synthesis of carotenoids. In the engineered *Y. lipolytica*, substrate inhibition was relieved by changing the specific properties of LC. The mutant LC^Y27R^ exhibited a complete loss of substrate inhibition while maintaining catalytic activity, resulting in the production β-carotene up to 2.38 g/l. Subsequently, through the regulation of metabolic flux, the engineered strain *Y. lipolytica* YLMA15 was able to produce 39.5 g/l β-carotene (Ma et al. 2022).

Substrate engineering has also been used to enable microorganisms to grow in one-carbon compounds such as CO_2_, formate, and methanol. Synthetic methylotrophic microorganisms have emerged as a landmark achievement in the field of synthetic biology. By functionally integrating methanol assimilation pathways into heterotrophic microbial hosts and subsequently refining their performance through adaptive laboratory evolution, artificial methylotrophs have been successfully established, including *E. coli* with a doubling time of 3.5 h (Nieh et al. 2024, Reiter et al. 2024) and methylotrophic *S. cerevisiae* with doubling time of 58.18 h (Guo et al. 2024). These engineered strains were able to utilize methanol as a carbon source for the biosynthesis of a diverse range of high-value chemicals such as lactic acid, PHB, itaconic acid, and *p*-aminobenzoic acid. Notably, citric acid production has been achieved at a titer of 1.0 g/l in a 5-l bioreactor using methanol as the sole carbon source. Furthermore, engineered strains utilizing formate and CO_2_ have shown remarkable potential. For instance, the formate-utilizing *E. coli* K4M not only exhibited a shorter doubling time than that of natural formate-utilizing strains, but also achieved the production of mevalonic acid up to 3.8 g/l. In addition, this strain was able to metabolize lignin hydrolysate as a substrate for producing mevalonic acid with a titer of 32.0 mg/l (Cowan et al. 2025). Progress has also been made in the development of microbial chassis capable of sustaining cell growth on CO_2_, representing a crucial advancement towards achieving carbon-neutral biomanufacturing (Gleizer et al. 2019). These advancements not only underscore the potential of substrate reprogramming to access unconventional feedstocks, but also establish a good foundation for expanding the spectrum of usable carbon sources (Reiter 2024).

Substrate engineering offers a comprehensive approach to broadening the range of substrate utilization and enhancing the cost-effectiveness of microbial production. A key benefit of this approach is its ability to exploit unconventional and sustainable feedstocks such as lignocellulosic biomass, C1 compounds, and industrial waste streams. However, the process of modifying microorganisms to efficiently break down and utilize diverse substrates typically requires addressing challenges, such as metabolic incompatibilities, enzyme limitations, and toxic intermediates. Through the systematic optimization of substrate degradation and utilization, substrate engineering facilitates the efficient conversion of a variety of low-cost and renewable feedstocks into valuable biochemicals (Silva et al. 2025). This approach not only mitigates the limitations associated with substrates in conventional bioprocesses but also improves the adaptability of microbial strains to complex or inhibitory raw materials. Consequently, substrate engineering is a crucial factor in promoting sustainable biomanufacturing and the valorization of alternative carbon sources (Ackdemir et al. 2022).

### Tolerance engineering

Tolerance engineering is an adjustment-control mode to avoid substantial viability loss by responsing to the stress conditions rapidly (Jiang et al. 2020), such as extreme pH, high osmotic pressure, harmful substrates, toxic metabolites, and so on, and adapt to these adverse environmental factors by metabolic engineering and synthetic biology strategies, such as evolutionary engineering, genome shuffling, random mutagenesis, and so on (Qi et al. 2019). Tolerance engineering can be applied for reinforcement of stress tolerance, diagnosis of stress response, selection of robust strains, and improvement of bioprocess economics. To realize these goals, four corresponding strategies have been developed to respond the cell-destructive damages (Tran and Zhao 2022): (i) inducing general or specific stress responses to improve major or specialized components; (ii) inducing detoxification mechanisms to convert toxic compounds into less toxic one, transport toxic chemicals out of cells, and produce protective metabolites to counteract toxic effects; (iii) inducing longer-term adaptive responses to overcome solvent toxicity; and (iv) inducing complex changes in transcriptional and protein levels to programmatically or systematically enhance cell tolerance to environmental stress.

The activation of general or specific stress responses requires the regulation of corresponding cellular mechanisms—either global or targeted—that function to repair or stabilize critical cellular structures and functions. This regulation ultimately enhances cell survival and growth in the face of adverse environmental conditions (Lin et al. 2013). A representative example is the enhancement of stress tolerance in *S. cerevisiae* through engineering the prokaryotic global regulator IrrE. Directed evolution was employed to modify the global transcriptional regulator IrrE from *Deinococcus radiodurans*, resulting in three variants that significantly improved yeast tolerance to ferulic acid and other aromatic inhibitors (FAPs). Transcriptomic and metabolomic analyses revealed that the engineered IrrE modulates multiple cellular defense systems to counteract FAP-induced stress, including the regulation of reactive oxygen species detoxification, energy metabolism, and membrane system stability. These modifications collectively enhanced the growth performance of *S. cerevisiae* under complex inhibitory conditions (Wang et al. 2020a).

Inducing detoxification mechanisms entails engineering cellular detoxification pathways to counteract the accumulation of toxic compounds. This can be achieved by expressing specific enzymes that convert toxic substances to nontoxic or less toxic forms, or by introducing or enhancing efflux systems to actively expel toxic molecules from the cell, thereby minimizing damage to proteins, membranes, or DNA (Lv et al. 2020). To construct a microbial cell factory for limonene production, the inherent toxicity of limonene has posed a significant challenge. Through transcriptomic analysis, 82 genes from *Y. lipolytica* were identified with the potential to enhance limonene tolerance and transport functions. Notably, the overexpression of protein YALI0F19492p resulted in an 8-fold increase in limonene production and a 3.2-fold enhancement in the rate of cell survival. Additionally, short-term adaptive laboratory evolution was employed to further augment limonene tolerance and production, and the underlying tolerance mechanisms were elucidated through analyses of cellular morphology and physiological characteristics (Li et al. 2021).

Inducing long-term adaptive responses focuses on the sustained or cumulative adaptation of cells to continuous stress. This typically requires the coordinated regulation of multiple genes, allowing cells to fundamentally reprogram their physiological state to cope with specific stressors (Wan et al. 2024). A good example of tolerance engineering is ethanol production. *S. cerevisiae* is widely acknowledged as the best producer for ethanol, but ethanol still shows a strong inhibition in cell growth, thus limiting its productivity. To meet the need of industrial applications, two yeast mutant libraries were created using the unmutated TATA-binding protein (SPT15) and its related factors (TAF25). Among these mutations, the best mutant STP15^F177S/Y195H/K218R^ not only increased ethanol tolerance, but also converted glucose to ethanol more efficiently (Alper et al. 2006).

Tolerance engineering serves as a multifaceted and effective approach for improving microbial resilience in the face of adverse environmental and industrial stress, such as toxic substances, extreme pH levels, and osmotic stress. It enables microorganisms to sustain viability and productivity in unfavorable conditions, thereby expanding the operational capacity of bioprocesses. However, the implementation of tolerance mechanisms can impose metabolic burdens, induce unintended regulatory effects, or interfere with the biosynthetic pathways, particularly when stress responses are consistently active. This methodology is independent on a singular mechanism but instead it incorporates a range of strategies for the activation of general or specific stress responses, the enhancement of detoxification pathways, the facilitation of adaptive evolution, and the reconfiguration of transcriptional networks (Guan and Liu 2020). These strategies enable microbial cells not only to endure stress but also to maintain efficient biosynthetic processes under such conditions. Consequently, tolerance engineering establishes a foundation for the creation of robust microbial platforms that can achieve high production in demanding bioprocess environments (Xu et al. 2022).

### Chassis engineering

Microbial chassis engineering refers to the systematic optimization and reconstruction of microbial hosts to enhance their genetic stability, metabolic activity, environmental adaptability, and capacity for product biosynthesis. Traditionally, chassis design relies on gene knockouts or overexpression to regulate precursor availability, cofactor supply, and product transport, resulting in thousands of high-performance strains (Calero and Nikel 2019). With the rapid advancement of synthetic biology, chassis engineering has evolved to the design of artificial cellular systems, enabling synthetic cells to serve as platforms for the production of value-added biochemicals. Consequently, the development of microbial chassis engineering can be characterized by the transition from model to nonmodel microorganisms and from natural to synthetic cells (Liu et al. 2020b).

Model microbial chassis engineering involves the use of metabolically well-characterized microorganisms with established genetic toolkits, allowing modular and system-level design strategies to enhance production efficiency (Wang et al. 2023a). A representative example is the improvement of xylose utilization in *S. cerevisiae* via a modular engineering approach. Five engineering strategies—promoter engineering, transcription factor regulation, biosensor construction, heterologous enzyme expression, and enzyme engineering—were applied to redesign various modules in central carbon metabolism at both genetic and enzymatic levels. These modifications significantly enhanced the conversion of xylose to acetyl-CoA derivatives, resulting in 3-hydroxypropionic acid production up to 7.46 g/l, which was a 4.7-fold increase compared to that of the initially optimized strain (Li et al. 2025b).

**Figure 9.**
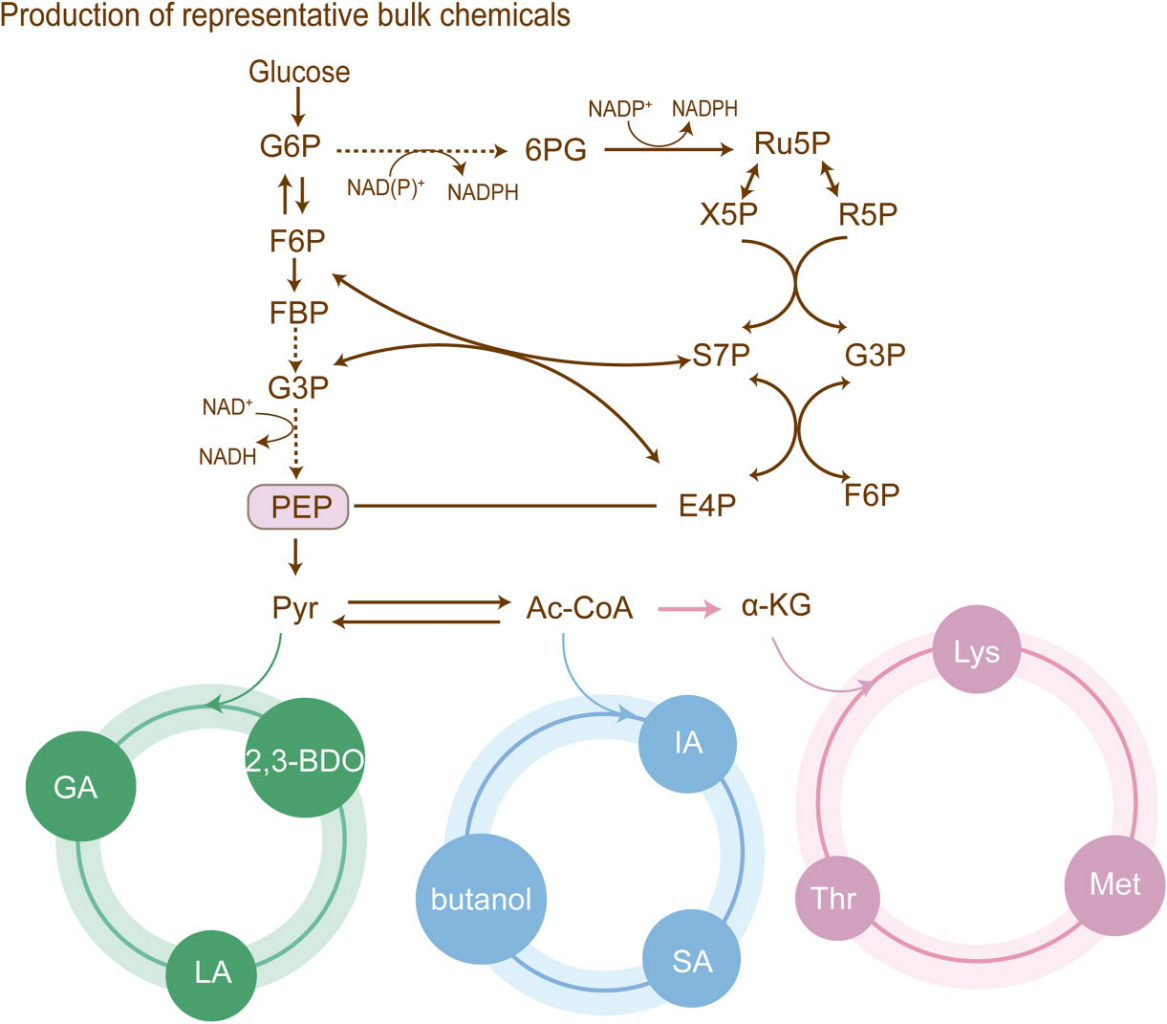
Production of representative bulk chemicals. G6P, glucose-6-phosphate; F6P, fructose-6-phosphate; FBP, fructose bisphosphate; G3P, glycerol-3-phosphate; PEP, phosphoenolpyruvate; Pyr, pyruvic acid; Ac-CoA, acetyl-CoA; α-KG, α-ketoglutaric acid; E4P, erythrose-4-phosphate; S7P, Sedoheptulose 7-phosphate; X5P, xylulose 5-phosphate; R5P, ribose-5-phosphate; Ru5P, ribulose-5-phosphate; 6PG, 6-phosphogluconate; GA, glycolaldehyde; LA, lactic acid; 2,3-BDO, 2,3-Butanediol; IA, itaconic acid; SA, succinic acid; Lys, lysine; Thr, threonine; and Met, methionine.

Nonmodel microbial chassis engineering aims to develop microorganisms with unique metabolic capabilities but limited genetic tools, enabling them to support complex biosynthetic pathways (Liu et al. 2020c). A representative example is the use of the nonconventional yeast *Y. lipolytica* to establish a robust platform for succinate production. By rewiring the TCA and glyoxylate cycles, deleting the competing pathways, and introducing a heterologous transporter, the engineered strain *Y. lipolytica* ST-109 efficiently converted glycerol to succinate, reaching 130.99 g/l without external pH control. Subsequent adaptive evolution under increasing glucose concentrations further improved strain robustness, leading to the production of 106.68 g/l succinate from glucose (Sun et al. 2025). This case illustrates the potential of nonconventional microbial hosts to supplement conventional chassis, offering high productivity alongside unique metabolic and physiological benefits(Fig. 9).

Artificial cell chassis engineering signifies a transition from natural biological systems to entirely synthetic frameworks, focusing on the reconstruction of cellular functions in nonnatural environments. A representative example is the creation of an artificial yeast chromosome, representing significant advancement in designing and constructing eukaryotic genomes. Using the specialized computer-aided design platform BioStudio (Richardson et al. 2017), researchers systematically redesigned chromosome II of *S. cerevisiae* to incorporate functional features, facilitating controllable bidirectional DNA recombination (Shen et al. 2016). Subsequently, an *I-SceI*-mediated chromosomal integration strategy was developed, wherein the chromosome was partitioned into DNA fragments of variable lengths for facilitating efficient stepwise assembly. However, high-throughput sequencing of the assembled strain identified two copy number variations. To address these structural irregularities, a streamlined and marker-free repair method was established, in which *I-SceI*-induced double-strand breaks triggered homologous recombination to correct tandem duplications, thus obtaining a strain harboring the accurately constructed synthetic chromosome (Parenteau et al. 2008). Phenotypic assessments demonstrated that, under 17 stress conditions, the synII strain exhibited growth characteristics similar to the wild-type *S. cerevisiae* BY4741, showing no apparent deficiencies in chromosomal replication, segregation, or cell-cycle progression. Multi-omics analyses further confirmed that the synII strain displayed robust biological functionality comparable to the native strain across various levels, including transcriptomic, proteomic, metabolomic, and lipidomic profiles, while also enhancing processes associated with protein translation. The successful construction of synII strain not only confirms the feasibility and stability of synthetic eukaryotic genomes but also establishes solid groundwork for the future creation of synthetic yeast strains with diverse applications in industries, medicine, environment, energy, and agriculture (Shen et al. 2017).

Microbial chassis engineering provides a foundational strategy for constructing stable, efficient, and scalable cell factories. This approach facilitates the development of customized hosts with minimal metabolic burden, thereby improving genetic robustness, and high compatibility with synthetic circuits. However, the design of universally adaptable chassis remains challenging, due to the context-specific interplay between genetic parts and host physiology, particularly in nonmodel organisms. By evolving from model to nonmodel microorganisms and advancing toward synthetic cellular systems, it transcends the limitations of traditional metabolic control, equipping hosts with enhanced biosynthetic capacity and environmental adaptability. With the integration of omics technologies, artificial intelligence, and modular design, chassis engineering is poised to drive the next generation of complex and intelligent synthetic biological systems, offering a key avenue for high-performance microbial platform development (Komera et al. 2023).

## Conclusions and perspectives

With the continuous integration of synthetic biology and systems metabolic engineering, the design and reconstruction of microbial cell factories are advancing toward a new paradigm characterized by hierarchical architecture, systemic coordination, and intelligent optimization. This review systematically summarizes five levels of metabolic reprogramming strategies-namely parts, pathways, networks, genomes, and cells, offering insights for the third wave of metabolic engineering. By integrating representative case studies, the versatility, performance, and efficiency of each hierarchical strategy are discussed in enhancing the titer, yield, and productivity of target compounds. Based on this, a cross-scale conceptual framework for hierarchical metabolic engineering provides a theoretical foundation and strategic roadmap for the development of next-generation microbial cell factories with high precision, efficiency, and predictability. However, their practical implementation is hindered by three main challenges: the inefficiency of rational design in managing complex and nonlinear biological systems, the limited adaptability of standardized strategies to nonmodel organisms, and the absence of integrated and automated platforms to support high-throughput Design–Build–Test–Learn (DBTL) cycles. Thus, future efforts will likely focus on leveraging machine learning to improve design precision, expanding hierarchical frameworks to nonmodel microbial hosts with unique metabolic capabilities, and integrating standardized hierarchical strategies into automated biofoundry platforms for scalable and programmable strain development.

With the explosion of multi-omics data and rapid advances in computational biology, machine learning (ML) has emerged as a critical enabler for transitioning metabolic engineering from an empirical trial-and-error model to a data-driven, rational design paradigm. In hierarchical metabolic engineering, ML is expected to play increasingly pivotal roles. At part level, ML models can be used to predict the function of regulatory elements such as promoters, RBS, and catalytic residues (Volk et al. 2020). At pathway level, graph neural networks and generative deep learning models facilitate de novo pathway design and bottleneck identification (Goshisht 2024). At network and cell levels, ML enables modeling, simulation, and intervention of complex regulatory networks (Yang et al. 2024). A representative example is the application of ML to overcome the long-standing challenge of substrate promiscuity and byproduct formation in chalcone synthase (CHS). To tackle this challenge, a library of 243 EbCHS variants was constructed, and an XGBoost model was developed by incorporating one-hot encoding, ESM-2 embeddings, and physicochemical characteristics utilizing data on single- and double-mutations. This approach facilitated the precise prediction of multimutation combinations, leading to the identification of ten promising triple- and quadruple mutants from more than 10^3^ potential options. Guided by these predictions, the EbCHS^F168Y/S211G/C344A^ mutant exhibited 92-fold enhancement in selectivity towards phloretin/naringenin, resulting in phloretin production up to 132.85 mg/l (*p*-coumaric acid pathway) and 164.29 mg/l (phloretic acid pathway). Structural analysis further revealed that the three mutations cooperatively reshaped the active site to mitigate off-target binding and reinforce hydrogen bond networks for the desired substrate (Li et al. 2025a). This case demonstrates the capacity of ML to facilitate efficient navigation of complex sequence-activity landscapes in a data-driven “design–build–test–learn” cycle. Furthermore, integration with Bayesian optimization and reinforcement learning algorithms has enabled DBTL cycles to evolve into closed-loop intelligent design systems, significantly accelerating strain construction and pathway integration (Volk 2023). Nevertheless, critical challenges still remain, including limited data quality, poor model interpretability, and insufficient biological compatibility of algorithms. Future efforts should focus on establishing deeper synergies between AI-based design and high-throughput experimental platforms.

To date, hierarchical metabolic engineering has primarily been applied in model microorganisms such as *E. coli* and *S. cerevisiae*. However, nonmodel microorganisms—owing to their unique metabolic traits, environmental adaptability, and capacity for natural product biosynthesis—are increasingly recognized as valuable chassis for emerging biomanufacturing applications (Papadaki et al. 2025). Despite their potential, nonmodel species still face considerable barriers, such as a lack of genetic toolkits, incomplete metabolic maps, and underdeveloped regulatory systems (Cheng et al. 2023). Therefore, future hierarchical strategies can prioritize the development of versatile chassis engineering methods for nonmodel strains, to develop high-throughput libraries of regulatory elements, interoperable modular designs, and network inference-based pathway mining (Volke et al. 2023, Kozaeva et al. 2024, Federici et al. 2025). Moreover, the application of AI-driven cross-species modeling with few-shot and transfer learning algorithms is essential for expanding the design space of nonmodel hosts, thereby enabling their deployment in specialized fields such as green chemistry, extremophile enzyme production, and biomineralization.

Biofoundry development amplifies the relevance of hierarchical metabolic engineering, particularly through its emphasis on standardization and modularization. By developing standardized databases of genetic parts, pathway modules, and regulatory frameworks, hierarchical strategies can substantially enhance the design accuracy and assembly throughput of biofoundry workflows (Kong et al. 2025). In addition to physical modularization, it is crucial to establish a knowledge-sharing engineering framework based on interoperable standards and digital infrastructure to facilitate the reproducible, traceable, and reusable transfer of genetic designs among laboratories. This can be achieved through initiatives, such as the Standard European Vector Architecture, the Synthetic Biology Open Language, and the Inventory of Composable Elements repositories (Martínez-García et al. 2023). Future integration of automated construction and testing platforms with AI-assisted design algorithms will enable fully closed-loop control, from part selection and pathway optimization to genome editing and whole-cell phenotyping (Jiang et al. 2022). In parallel, the hierarchical framework may serve as a foundation for codifying engineering principles and protocols in synthetic biology, facilitating interoperability and reproducibility across laboratories and industries. Ultimately, the convergence of hierarchical integration, digitalized workflows, and multidimensional optimization in biofoundry platforms is expected to become a cornerstone of the next-generation metabolic engineering paradigm, driving advances in precision manufacturing, bioeconomy development, and the broader transition toward Industry 4.0 in the life sciences (Lawson et al. 2021).
